# From Fatty Acid Precursors to Off-Odor in Canned Antarctic Krill: Matrix-Dependent Odorant Expression Under Retort Sterilization Conditions

**DOI:** 10.3390/foods15173053

**Published:** 2026-08-28

**Authors:** Peizi Sun, Songyi Lin, Yuting Tan, Yajie Qin, Xiaobin Bi, Dongmei Li

**Affiliations:** 1 School of Food Science and Technology, National Engineering Research Center of Seafood, Dalian Polytechnic University, Dalian 116034, China; 2 Engineering Research Center of Seafood of Ministry of Education of China, Dalian 116034, China; 3 Collaborative Innovation Center of Seafood Deep Processing, Dalian Polytechnic University, Dalian 116034, China; 4 State Key Laboratory of Marine Food Processing and Safety Control, Dalian Polytechnic University, Dalian 116034, China

**Keywords:** Antarctic krill, fatty acid oxidation, thermal oxidation models, odor activity value, matrix effect

## Abstract

Off-odor development during retort sterilization limits the sensory quality of canned Antarctic krill, but measurements in whole krill cannot distinguish intrinsic fatty acid oxidation from matrix-dependent odor expression. To separate these effects, six major fatty acids (C14:0, C16:0, C16:1, C18:1, eicosapentaenoic acid [EPA], and docosahexaenoic acid [DHA]) were examined in a three-tier thermal oxidation model comprising fatty acid-only, lipid-depleted krill matrix, and complete krill meat systems heated at 115 °C for 36 min. Volatile profiles were characterized by gas chromatography–ion mobility spectrometry (GC–IMS) and headspace solid-phase microextraction–gas chromatography–mass spectrometry (HS-SPME–GC–MS), together with peroxide value (POV), thiobarbituric acid-reactive substances (TBARS), odor activity values (OAVs), sensory evaluation, and exploratory cross-tier correlation analysis. In the fatty acid-only model, EPA and DHA showed the highest POVs (5.26 ± 0.53 and 4.96 ± 0.57 meq/kg), whereas C16:1 and C18:1 produced broader volatile profiles. After matrix incorporation, these patterns changed: C18:1 supplementation was associated with a pronounced aldehydic profile, whereas DHA- and EPA-supplemented complete krill meat showed the highest TBARS values (3.37 ± 0.40 and 3.12 ± 0.37 mg MDA/kg) and high fishy odor scores (6.21 and 5.95). Total OAV was not significantly associated with fishy or oxidized odor, whereas (Z)-4-heptenal, (Z)-1,5-octadien-3-ol, 1-penten-3-ol, and trimethylamine showed closer sensory associations. Thus, intrinsic oxidation potential did not directly predict off-odor intensity; sensory deterioration was more closely related to the matrix-dependent expression of selected potentially odor-active compounds than to fatty acid unsaturation or total OAV.

## 1. Introduction

Retort sterilization is essential for ensuring the microbial safety and shelf stability of canned Antarctic krill (*Euphausia superba*), but the associated thermal load can substantially alter its odor quality. Thermal treatment produces distinct volatile fingerprints and sensory characteristics in Antarctic krill [1], while marked changes in volatile composition occur across different stages of krill canning [2]. Sterilization intensity further affects the abundance and distribution of alcohols, aldehydes, ketones, esters, and furans in canned krill [3]. Quantitative evidence also shows that steaming increased nonanal from 2.23 ± 0.06 to 8.14 ± 1.26 μg/kg, with nonanal and 1-octen-3-ol associated with fatty acid degradation [4]. These findings establish thermal processing as an important driver of volatile changes in Antarctic krill. However, measurements made in whole krill products cannot distinguish the intrinsic contribution of individual fatty acid precursors from the effects imposed by the surrounding matrix.

Antarctic krill lipids are rich in polar lipids and long-chain n-3 polyunsaturated fatty acids, particularly eicosapentaenoic acid (EPA) and docosahexaenoic acid (DHA) [5,6]. During thermal oxidation, hydrogen abstraction initiates lipid radical formation, followed by oxygen addition and the formation of peroxyl radicals and hydroperoxides. Thermal decomposition of hydroperoxides generates alkoxyl radicals that undergo cleavage and secondary reactions, producing aldehydes, ketones, alcohols, furans, hydrocarbons, and other volatile products [7]. The number and position of double bonds affect both oxidation susceptibility and the distribution of cleavage products, while the sensory relevance of the resulting volatiles also depends on their odor thresholds and odor characteristics [7,8]. Different fatty acids can therefore produce distinct volatile patterns and sensory effects under comparable heating conditions [8,9]. Examining representative fatty acids individually is necessary to establish their intrinsic oxidation and volatile-forming behavior before these patterns can be interpreted in a complex krill matrix.

The krill matrix adds a further level of complexity. Fishy and oxidation-related odors in aquatic products can arise from lipid oxidation, protein degradation, amine formation, sulfur-containing reactions, and interactions among these pathways [10]. Proteins, endogenous lipids, and other non-volatile constituents can also influence volatile retention, partitioning, headspace release, and secondary reactions [11]. Consequently, headspace abundance cannot be interpreted as a direct measure of fatty acid oxidation alone. Controlled lipid systems have been used to relate fatty acid changes to lipid oxidation and volatile development in aquatic foods [12], and heated seafood flavor formulations containing EPA, DHA, or fish oil have produced compounds such as hexanal, 4-heptenal, and 2,4-heptadienal [9]. These approaches establish precursor-related volatile chemistry, but separating intrinsic fatty acid behavior from its expression in krill matrices requires the same precursors to be examined across matrices of increasing complexity under a common thermal condition.

The fatty acid composition of raw Antarctic krill was therefore first determined, and six representative fatty acids—C14:0, C16:0, C16:1, C18:1, EPA, and DHA—were selected to cover the major saturated, monounsaturated, and long-chain n-3 polyunsaturated fatty acids. A three-tier thermal oxidation model system was then constructed, comprising fatty acid-only models (FA-only), lipid-depleted krill matrices supplemented with individual fatty acids (coded as LFK + FA), and complete krill meat matrices supplemented with individual fatty acids (KM + FA). All three model tiers were subjected to the same retort treatment. The FA-only models were used to establish intrinsic precursor-specific oxidation and volatile-forming behavior, whereas the LFK + FA and KM + FA models were used to examine how increasing matrix complexity altered the expression of these precursor-associated volatile patterns. The working hypothesis was that fatty acid structure determines the initial oxidation and volatile-forming potential, while the krill matrix modifies how these precursor-associated signals are ultimately expressed.

Off-odor development was operationally evaluated as increased fishy and oxidized odor intensities accompanied by decreased overall odor acceptability. Gas chromatography–ion mobility spectrometry (GC–IMS) and headspace solid-phase microextraction–gas chromatography–mass spectrometry (HS-SPME-GC–MS) were combined with peroxide value (POV), thiobarbituric acid-reactive substances (TBARS), odor activity values (OAVs), sensory evaluation, and correlation analysis. The study focused on three questions: (i) how the six representative fatty acids differ in intrinsic oxidation and volatile-forming behavior; (ii) how these precursor-associated volatile patterns change after incorporation into lipid-depleted and complete krill matrices; and (iii) which oxidation-related, potentially odor-active compounds are most closely associated with fishy odor, oxidized odor, and overall odor acceptability under retort sterilization conditions.

## 2. Materials and Methods

### 2.1. Materials and Chemicals

Frozen Antarctic krill (*Euphausia superba*) meat was supplied by Dalian Liaoyu Fishery Group Co., Ltd. (Dalian, China). The krill were harvested from the Antarctic FAO 48.1 fishing area in May 2025, peeled on board, and immediately frozen at −30 °C. The samples were transported to the laboratory under cold-chain conditions in August 2025, divided into approximately 600 g portions, vacuum-packaged, and stored at −20 °C until use.

Myristic acid (C14:0, ≥95%), palmitic acid (C16:0, ≥95%), palmitoleic acid (C16:1, ≥95%), oleic acid (C18:1, ≥95%), eicosapentaenoic acid (EPA, ≥95%), docosahexaenoic acid (DHA, ≥95%), and 2,4,6-trimethylpyridine (internal standard) were purchased from Shanghai Macklin Biochemical Co., Ltd. (Shanghai, China). n-Alkane standards (C4–C9 and C7–C30, ≥97%) were purchased from Shanghai Aladdin Biochemical Technology Co., Ltd. (Shanghai, China). Sodium chloride, chloroform, methanol, hexane, isopropanol, boron trifluoride–methanol solution, sodium hydroxide–methanol solution, ferric chloride, ammonium thiocyanate, trichloroacetic acid, thiobarbituric acid, and other reagents were purchased from Shanghai Macklin Biochemical Co., Ltd. unless otherwise stated. All chemicals were of analytical grade or higher.

### 2.2. Preparation of Lipid-Depleted Antarctic Krill Matrix

The lipid-depleted Antarctic krill matrix (sample code LFK) was prepared using a modified hexane/isopropanol lipid-extraction procedure based on Hara and Radin [13] and Showman et al. [14]. Freeze-dried Antarctic krill powder was extracted three times with hexane/isopropanol (3:2, *v*/*v*) at a solid-to-solvent ratio of 1:6 (g/mL). For each extraction, the mixture was dispersed with fresh solvent for 15 min, allowed to stand for 10 min, and the lipid-containing supernatant was removed.

After the final extraction, the residue was centrifuged at 10,000× *g* for 8 min and washed with hexane at a ratio of 1:4 (g/mL), followed by centrifugation at 10,000× *g* for 6 min. The washing step was repeated when necessary until the supernatant became clear. The solvent-extracted residue was spread as a thin layer (<5 mm) on glass trays and dried in a fume hood under adequate ventilation at room temperature for 16 h with periodic turning. Drying was continued until no perceptible residual solvent odor remained. The dried LFK was ground, homogenized, sealed, and stored at −20 °C. Before use, the LFK powder was hydrated with deionized water at a ratio of 1:4 (*w*/*w*) and equilibrated at 4 °C for 2 h. Because residual lipid content was not independently quantified after extraction, the solvent-extracted krill matrix is referred to as a lipid-depleted krill matrix rather than a lipid-free krill matrix throughout this study.

### 2.3. Fatty Acid Composition Analysis

Fatty acid composition was determined before model construction to guide the selection of representative fatty acid precursors. Fatty acid methyl esters were prepared according to GB 5009.168-2016 [15] with minor modifications. Briefly, 5 mg of lipid sample was mixed with 200 μL of tridecanoyl glyceride internal standard solution (1 mg/mL in chloroform). Then, 2 mL of 0.5 mol/L sodium hydroxide–methanol solution was added, and the mixture was heated at 80 °C for 5 min. Subsequently, 2 mL of 14% (*w*/*w*) boron trifluoride–methanol solution was added, and the reaction was continued for 2 min. After cooling, fatty acid methyl esters were extracted with 1.5 mL of hexane and filtered through a 0.22 μm membrane before analysis.

Fatty acid methyl esters were analyzed using an Agilent 7890B gas chromatograph (Agilent Technologies Inc., Santa Clara, CA, USA) equipped with a flame ionization detector and an SP-2560 capillary column (100 m × 0.25 mm, 0.20 μm; Supelco, Bellefonte, PA, USA). The oven temperature was held at 140 °C for 5 min, increased to 180 °C at 4 °C/min and held for 20 min, and then increased to 240 °C at 4 °C/min and held for 15 min. Nitrogen was used as the carrier gas. The injector and detector temperatures were both set at 260 °C. Fatty acids were identified by comparison with authentic standards and expressed as percentages of total fatty acids.

### 2.4. Model System Preparation and Thermal Treatment

The experiment comprised 20 treatments organized within a three-tier thermal oxidation model system: six fatty acid-only treatments (FA-only; C14:0, C16:0, C16:1, C18:1, EPA, and DHA), seven lipid-depleted krill matrix treatments consisting of an LFK blank and six fatty acid-supplemented groups (LFK + FA), and seven complete krill meat treatments consisting of a KM blank and six fatty acid-supplemented groups (KM + FA). The complete sample coding scheme is provided in Table S1.

For the LFK + FA treatments, 29.85 g of hydrated lipid-depleted krill matrix was mixed with 0.15 g of the corresponding fatty acid, giving a total mass of 30.00 g and a fatty acid supplementation level of 5.0 g/kg. For the KM + FA treatments, 29.85 g of homogenized krill meat was mixed with 0.15 g of the corresponding fatty acid at the same supplementation level. The LFK and KM groups were prepared as matrix blanks without fatty acid addition.

All matrix mixtures were packed in heat-resistant vacuum pouches, thoroughly mixed, vacuum-sealed, flattened, and heated at 115 °C for 36 min in a counter-pressure steam retort (ZM-100; Guangzhou Biaoqi Packaging Equipment Co., Ltd., Guangzhou, China). After heating, the samples were immediately cooled in ice water for 20 min. Separate independently prepared units were used for volatile analysis, lipid oxidation analysis, and sensory evaluation. Three independently prepared and heated units were used for each treatment and were regarded as independent experimental replicates.

For the FA-only model, the amount of each fatty acid was matched to the fatty acid loading used in the corresponding LFK + FA and KM + FA systems, equivalent to 5 mg of fatty acid per gram of matrix-equivalent sample (5.0 g/kg). Accordingly, 15 mg of fatty acid was used for GC–IMS analysis, corresponding to a 3.0 g matrix sample; 25 mg was used for HS-SPME-GC–MS analysis, corresponding to a 5.0 g matrix sample; 10 mg was used for POV determination, corresponding to a 2.0 g matrix sample; 25 mg was used for TBARS analysis, corresponding to a 5.0 g matrix sample; and 15 mg was used for sensory evaluation, corresponding to a 3.0 g matrix sample. Each FA-only sample was subjected to the same thermal treatment at 115 °C for 36 min and immediately cooled in ice water for 20 min. No NaCl solution was added to the FA-only samples.

Although fatty acid precursor loading was matched across the three model tiers for the corresponding analyses, the FA-only and matrix-containing systems differed substantially in sample composition and headspace partitioning behavior. Cross-tier interpretation therefore focused on precursor-dependent patterns, matrix modulation, and the occurrence and relative prominence of individual volatile compounds rather than direct ranking of absolute total VOC concentrations or total OAV magnitudes among the three model systems.

After cooling, each independently heated pouch allocated to volatile analysis was gently kneaded for 30 s before opening. For GC–IMS analysis, three 3.0 g aliquots were transferred into 20 mL headspace vials without salt addition. For HS-SPME-GC–MS analysis, three 5.0 g aliquots were transferred into 20 mL headspace vials, followed by 5.0 mL of saturated NaCl solution and 20 μL of 2,4,6-trimethylpyridine internal standard solution (50 mg/L).

### 2.5. GC–IMS Analysis

GC–IMS analysis was performed using a FlavourSpec^®^ system (G.A.S., Dortmund, Germany) according to previously reported methods for canned Antarctic krill with modifications [2,3]. For the LFK and KM systems, 3.00 g of sample was placed in a 20 mL headspace vial. For the FA-only model, 15 mg of the corresponding fatty acid was used, matching the amount of fatty acid supplemented into 3.00 g of matrix sample at 5.0 g/kg. Samples were equilibrated at 80 °C for 20 min with stirring at 500 rpm. The syringe temperature was set at 85 °C, and 1000 μL of headspace gas was injected into an MXT-WAX column (30 m × 0.53 mm, 1 μm).

The column temperature was 45 °C. High-purity nitrogen (≥99.999%) was used as the carrier gas. The carrier gas program was 2 mL/min for 2 min, increased to 10 mL/min within 10 min, increased to 100 mL/min within 20 min, and then maintained at 100 mL/min for 30 min. The drift gas flow rate was 150 mL/min. n-Alkanes (C4–C9) were used for retention index calculation. Volatile compounds were tentatively identified by comparing retention indices and drift times with the GC × IMS Library Search software (v1.0.3, G.A.S., Dortmund, Germany).

### 2.6. HS-SPME-GC–MS Analysis

HS-SPME-GC–MS analysis was performed using an Agilent 7890B–5977B GC–MS system (Agilent Technologies Inc., Santa Clara, CA, USA) according to a previously reported method with modifications [3]. For the LFK and KM systems, each 20 mL headspace vial contained 5.00 g of sample, 5.0 mL saturated NaCl solution, and 20 μL of 2,4,6-trimethylpyridine internal standard solution (50 mg/L). For the FA-only model, each vial contained 25 mg of the corresponding fatty acid and 20 μL of the same internal standard solution, matching the amount of fatty acid supplemented into 5.00 g of matrix sample at 5.0 g/kg; no NaCl solution was added. The vial was equilibrated at 80 °C for 20 min, and volatiles were extracted with a DVB/CAR/PDMS fiber (50/30 μm) at 80 °C for 40 min. The fiber was desorbed in the GC injection port at 250 °C for 20 min.

Volatiles were separated on a DB-WAX capillary column (30 m × 250 μm × 0.25 μm). The oven temperature program was as follows: 35 °C initially; increased to 150 °C at 3 °C/min; increased to 240 °C at 10 °C/min; and finally increased to 250 °C at 15 °C/min. High-purity nitrogen was used as the carrier gas at a constant flow rate of 15 mL/min. The MS conditions were as follows: electron impact ionization energy, 70 eV; and mass scan range, *m*/*z* 30–500.

Linear retention indices were calculated using n-alkanes (C7–C30) as reference standards. Volatile compounds were tentatively annotated by comparing their mass spectra with the NIST 14 library and their experimental retention indices with literature values. Only annotations supported by consistent mass-spectral and retention-index evidence were retained. Compounds were considered reproducibly detected when present in at least two of three independent preparation/heating replicates. Relative concentrations of volatile organic compounds (VOCs) were estimated by single-internal-standard semi-quantification, expressed as μg/kg of sample, and calculated using Equation (1):

(1) $$C_{i}\left(\mu g/\mathrm{kg}\right)\;=\;\frac{A_{i}\;\times \;C_{s}\;\times \;V_{s}}{A_{s}\;\times \;m}\;\times \;1000$$

where (*C_i_*) is the relative concentration of the volatile compound (μg/kg), (*A_i_*) is the peak area of the volatile compound, (*C_s_*) is the mass concentration of the internal standard (μg/mL), (*V_s_*) is the volume of the internal standard solution (mL), (*A_s_*) is the peak area of the internal standard, and (m) is the sample mass (g). For FA-only samples, m was the actual fatty acid mass (0.025 g); for LFK and KM samples, m was 5.00 g.

### 2.7. Odor Activity Value Calculation

Odor activity values (OAVs) were calculated to evaluate the potential sensory relevance of volatile compounds. OAV was calculated using Equation (2):

(2) $$\mathrm{OAV}\;=\;\frac{C_{i}}{T_{i}}$$

where (*C_i_*) is the relative concentration of the volatile compound in the sample and (*T_i_*) is the odor threshold of the corresponding compound obtained from the literature [16,17,18,19,20,21]. Compounds with OAV > 1 were considered potentially odor-active compounds and potential contributors to the overall odor profile. Because VOC concentrations were obtained by single-internal-standard semi-quantification rather than compound-specific calibration, OAVs were interpreted as comparative indicators of potential odor relevance rather than absolute sensory contributions.

### 2.8. Peroxide Value

Peroxide value (POV) was determined using the ferric thiocyanate method described by Vareltzis et al. [22] with minor modifications. For the LFK and KM systems, 2.00 g of sample was used. For the FA-only model, 10 mg of each fatty acid was used, matching the amount of fatty acid supplemented into 2.00 g of matrix sample at 5.0 g/kg. Samples were homogenized with 15 mL of chloroform/methanol (2:1, *v*/*v*) for 30 s using a T25 homogenizer (IKA-Werke GmbH & Co. KG, Staufen, Germany). Then, 3 mL of 5 g/L NaCl solution was added, and the mixture was centrifuged at 3820× *g* for 5 min using an H1850R refrigerated centrifuge (Hunan Xiangyi Laboratory Instrument Development Co., Ltd., Changsha, China). A 5 mL aliquot of the lower phase was mixed with 5 mL of chloroform/methanol (2:1, *v*/*v*), followed by the addition of 25 μL FeCl_2_ solution and 25 μL ammonium thiocyanate solution (300 g/L). After vortexing for 5 s and standing at 25 °C for 5 min, absorbance was measured at 500 nm using a UV-1000 UV–visible spectrophotometer (Shanghai Tianmei Scientific Instrument Co., Ltd., Shanghai, China). A standard curve was prepared using reduced iron powder: *y* = 0.0422*x* + 0.0194 (*R*^2^ = 0.9976).

POV was calculated using Equation (3):

(3) $$\mathrm{POV}\;=\;\frac{x}{55.84\;\times \;W\;\times \;2\;\times \;f}$$

where (*x*) is the iron content calculated from the standard curve (μg), (*W*) is the sample mass (g), 55.84 is the atomic mass of iron, 2 is the conversion factor, and (*f*) is the sampling fraction of the lower phase used for color development. In this study, (*f*) was 0.333. For FA-only samples, W was defined as the corresponding matrix-equivalent sample mass (2.00 g), rather than the actual fatty acid mass, based on the unified loading of 5 mg fatty acid per gram of matrix-equivalent sample. POV was expressed as meq/kg sample.

### 2.9. TBARS Analysis

TBARS were determined according to Tan et al. [23] with minor modifications. For the LFK and KM systems, 5.00 g of sample was extracted with 25 mL of 75 g/L trichloroacetic acid solution. For the FA-only model, 25 mg of each fatty acid was used, matching the amount of fatty acid supplemented into 5.00 g of matrix sample at 5.0 g/kg, and was processed using the same extraction volume. After homogenization for 1 min using a T25 homogenizer (IKA-Werke GmbH & Co. KG, Staufen, Germany) and filtration, the filtrate was mixed with 0.02 mol/L thiobarbituric acid solution at a ratio of 1:1 (*v*/*v*), heated at 100 °C for 40 min, cooled, and centrifuged at 8000× *g* for 10 min using an H1850R refrigerated centrifuge (Hunan Xiangyi Laboratory Instrument Development Co., Ltd., Changsha, China). Absorbance was measured at 532 and 600 nm using an Infinite M200 multimode microplate reader (Tecan, Männedorf, Switzerland).

TBARS was calculated using Equation (4):

(4) $$\mathrm{TBARS}\;=\;\frac{\left(A_{532}\;-\;A_{600}\right)\;\times \;72.06}{155\;\times \;m}\;\times \;100$$

where (*A*_532_) and (*A*_600_) are the absorbance values at 532 and 600 nm, respectively; 72.06 is the molar mass of malondialdehyde (MDA) (g/mol); 155 is the molar absorption coefficient of the malondialdehyde–thiobarbituric acid complex; and (*m*) is the sample mass (g). For FA-only samples, m was defined as the corresponding matrix-equivalent sample mass (5.00 g), rather than the actual fatty acid mass, based on the unified loading of 5 mg fatty acid per gram of matrix-equivalent sample. TBARS values were expressed as mg MDA/kg sample.

### 2.10. Sensory Evaluation

Odor evaluation was performed by 30 trained panelists, including 15 females and 15 males aged 20–30 years. All panelists had experience in lipid-related odor evaluation and were trained according to GB/T 16291.1-2012 [24]. The evaluated attributes were fishy, fatty, oxidized, shrimp-like, and overall odor acceptability. Training comprised familiarization with the definitions and reference materials for the four descriptive odor attributes, practice in using the 0–9 scales, and repeated evaluation of representative samples. Consensus was established on the interpretation of the descriptive attributes, and panelists proceeded to formal evaluation after demonstrating satisfactory discrimination and repeatability during training. Overall odor acceptability was evaluated separately as a panel-rated overall response to the sample odor. Attribute definitions and reference materials are provided in Table S2. All panelists participated voluntarily and provided written informed consent before evaluation.

For the LFK and KM systems, 3.00 g of sample was placed in a 20 mL amber headspace vial. For the FA-only model, 15 mg of the corresponding fatty acid was used, matching the amount supplemented into 3.00 g of matrix sample at 5.0 g/kg. All vials were coded with random three-digit numbers. Panelists evaluated odor by gently rotating and sniffing each vial. Fishy, fatty, oxidized, and shrimp-like odor intensities were rated from 0 (not perceived) to 9 (extremely strong), whereas odor acceptability was rated from 0 (extremely unacceptable) to 9 (extremely acceptable). To limit olfactory fatigue, the 20 treatments were divided into four blocks of five samples and evaluated in four separate sessions. The order of the sessions and the presentation order of samples within each session were balanced and randomized across panelists. A minimum interval of 1 min was provided between samples, and panelists rested for at least 10 min between sessions. Each of the 30 panelists evaluated all 20 treatments once across the four sessions.

### 2.11. Data Processing and Statistical Analysis

All experiments were conducted in triplicate unless otherwise stated. Three independently prepared and heated units were treated as independent experimental replicates for each treatment. Technical measurements derived from the same heated unit were averaged before statistical analysis and were not regarded as independent observations.

Statistical analysis was performed using SPSS Statistics 26.0 (IBM Corp., Armonk, NY, USA), and results are expressed as mean ± standard deviation. For instrumental measurements, differences among treatments were evaluated by one-way analysis of variance followed by Tukey’s test, with statistical significance set at *p* < 0.05. For sensory data, each attribute was analyzed separately using a linear mixed-effects model, with treatment specified as a fixed effect and panelist specified as a random effect to account for repeated evaluations of different treatments by the same panelists. Statistical significance was set at *p* < 0.05.

Pearson correlation analysis was performed across the treatment means of all 20 groups as an exploratory cross-tier analysis of associations among oxidation indices, sensory attributes, total OAV, the number of compounds with OAV > 1, and selected potentially odor-active compounds. Because the FA-only and matrix-containing systems differed in sample composition, headspace partitioning, and VOC normalization basis, these pooled correlations were not interpreted as direct cross-model effect sizes or as evidence of causal relationships within individual model tiers. Principal component analysis (PCA), heatmap analysis, hierarchical cluster analysis (HCA), and correlation analysis were performed using VOCal 0.1.1 gamma, MetaboAnalyst 5.0, and OriginPro 2025, as appropriate for the corresponding datasets. Final figures were prepared using OriginPro 2025.

## 3. Results and Discussion

### 3.1. Fatty Acid Composition of Raw Antarctic Krill Meat and Selection of Representative Precursors

**Figure 1:**
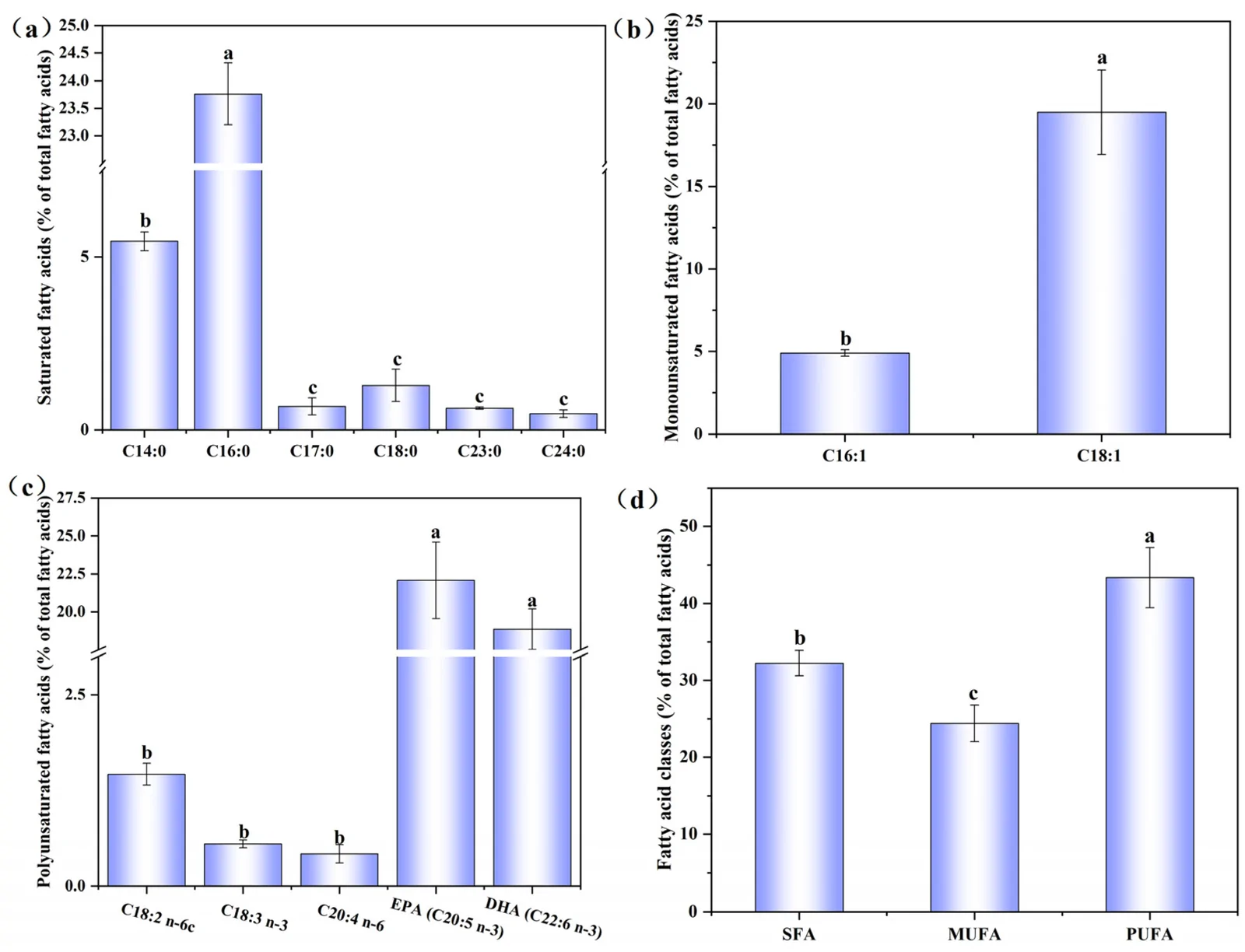
Fatty acid composition of raw Antarctic krill meat used for representative precursor selection. (**a**) Saturated fatty acids; (**b**) monounsaturated fatty acids; (**c**) polyunsaturated fatty acids; and (**d**) fatty acid class distribution. Values are expressed as % of total fatty acids. C18:1 represents total C18:1 isomers. Different lowercase letters indicate significant differences among fatty acids within the same panel (*p* < 0.05). The selected representative precursors for model construction were C14:0, C16:0, C16:1, C18:1, EPA (C20:5 n-3), and DHA (C22:6 n-3).

The fatty acid profile of raw Antarctic krill meat was first characterized to guide the selection of fatty acids for the thermal oxidation models (Figure 1). C16:0 was the most abundant individual fatty acid, accounting for approximately 23.7% of total fatty acids, followed by EPA (C20:5 n-3; 22.1%), C18:1 (19.4%), and DHA (C22:6 n-3; 18.9%). C14:0 and C16:1 accounted for approximately 5.5% and 4.9%, respectively. At the class level, PUFAs represented the largest fraction, followed by SFAs and MUFAs. This distribution agrees with the typical lipid characteristics of Antarctic krill, in which long-chain n-3 PUFAs, particularly EPA and DHA, are major fatty acid components [5,6].

Based on this profile, C14:0, C16:0, C16:1, C18:1, EPA, and DHA were selected for the model systems. Together, these six fatty acids accounted for approximately 94.5% of total fatty acids. They also differed substantially in degree of unsaturation: C14:0 and C16:0 contain no carbon–carbon double bonds, C16:1 and C18:1 each contain one, EPA contains five, and DHA contains six. The selected fatty acids therefore covered saturated, monounsaturated, and highly unsaturated marine n-3 fatty acids, with the number of double bonds ranging from 0 to 6.

These differences in unsaturation were expected to affect fatty acid oxidation during heating. Increasing unsaturation generally increases the availability of allylic and, particularly, bis-allylic hydrogen sites susceptible to abstraction and subsequent hydroperoxide formation [7,25]. However, the volatile products formed during oxidation also depend on double-bond position, the hydroperoxides formed, and their subsequent cleavage reactions. Thus, differences in unsaturation alone cannot fully predict the resulting volatile profile. The FA-only, LFK + FA, and KM + FA models were used to examine these fatty acids first as individual precursors and then within krill matrices, allowing their intrinsic oxidation behavior to be distinguished from changes in volatile expression associated with the surrounding matrix.

### 3.2. Differentiation of the Three-Tier Thermal Oxidation Models by GC–IMS Fingerprinting

**Figure 2:**
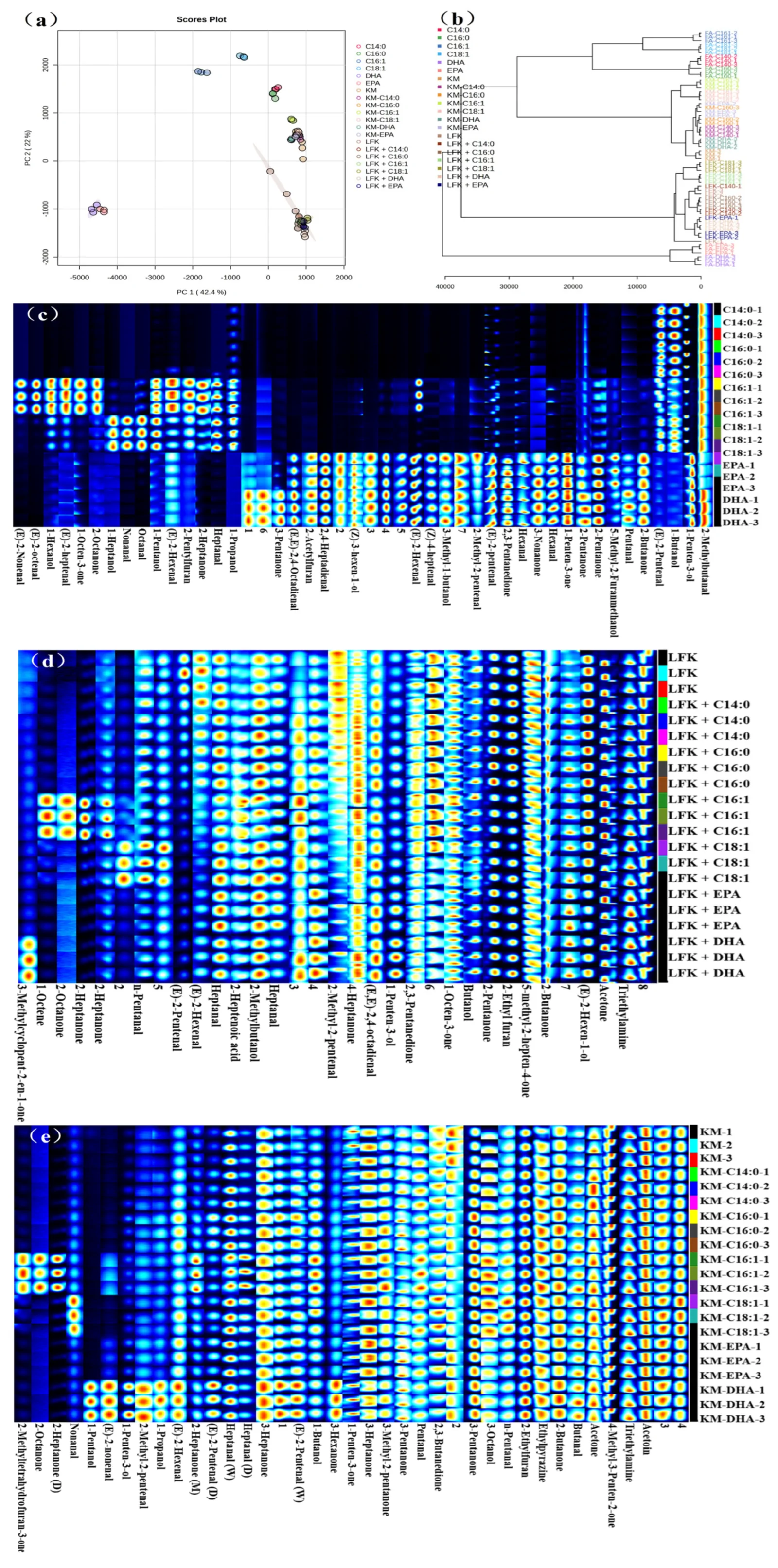
Gas chromatography–ion mobility spectrometry (GC–IMS) fingerprint analysis of volatile compounds generated from different fatty acid oxidation models. (**a**) Principal component analysis (PCA) score plot; (**b**) hierarchical clustering dendrogram; (**c**) gallery plot of the FA-only model; (**d**) gallery plot of the LFK + FA model; and (**e**) gallery plot of the KM + FA model. The gallery plots display the signal intensities of three independent replicates for each treatment, ordered by treatment group.

GC–IMS was used as a rapid, non-targeted approach to compare volatile fingerprints among the FA-only, LFK + FA, and KM + FA systems before compound-specific GC–MS analysis. The PCA score plot showed clear separation among the three model tiers (Figure 2a). PC1 and PC2 explained 42.3% and 21.6% of the total variance, respectively, accounting for 63.9% in combination. FA-only samples occupied a distinct region of the score plot, whereas LFK + FA and KM + FA samples shifted toward two separate matrix-related regions. Differences were also observed among fatty acid treatments within each model tier. These results indicate that both fatty acid type and matrix environment contributed to the variation captured by the GC–IMS fingerprints.

The HCA results were consistent with the PCA pattern (Figure 2b). FA-only, LFK + FA, and KM + FA samples formed separate clusters, whereas independent replicates within the same treatment clustered closely. The agreement between PCA and HCA further supported the consistency of the separation among the three model tiers.

The gallery plots further illustrated the differences in volatile fingerprints across the three model systems (Figure 2c–e). In the FA-only model, the six fatty acids produced clearly different signal patterns, with EPA and DHA showing more high-response regions than the saturated fatty acids. This pattern is consistent with the greater intrinsic oxidation susceptibility of highly unsaturated fatty acids. However, clear differences were also observed between C16:1 and C18:1 and between EPA and DHA, indicating that the resulting volatile fingerprints were not determined by the number of double bonds alone.

After incorporation into the lipid-depleted krill matrix, several prominent signal regions observed in the FA-only system became weaker or changed in relative intensity, and the overall fingerprints became more homogeneous. This change is consistent with matrix effects on volatile formation and/or headspace release rather than simple dilution. Proteins and other non-volatile constituents can influence volatile partitioning, retention, and release [11]. However, the GC–IMS fingerprints alone cannot distinguish changes in volatile formation from changes in headspace distribution.

The KM + FA model showed a further change in fingerprint pattern, with greater complexity than the FA-only and LFK + FA systems. Complete krill meat contains endogenous lipids, proteins, nitrogen-containing compounds, and other native constituents, providing a more complex chemical environment for volatile formation and release. The progressive changes from FA-only to LFK + FA and then to KM + FA show that the volatile patterns observed for individual fatty acids were increasingly modified after incorporation into krill matrices.

Overall, GC–IMS showed clear and consistent fingerprint differences among the three tiers of the thermal oxidation model. The results indicate that both fatty acid characteristics and the surrounding krill matrix contributed to the observed volatile fingerprint differences under the same thermal treatment. Because GC–IMS provides fingerprint-level discrimination rather than definitive compound-specific identification and quantification [26,27], HS-SPME-GC–MS was subsequently used to examine the volatile compounds underlying these differences.

### 3.3. Precursor-Specific Volatile Signatures in the FA-Only Model

The FA-only model was used to characterize the volatile profiles of the six individual fatty acids before krill-derived matrices were introduced, providing a precursor-level reference for comparison with the LFK + FA and KM + FA systems.

**Figure 3:**
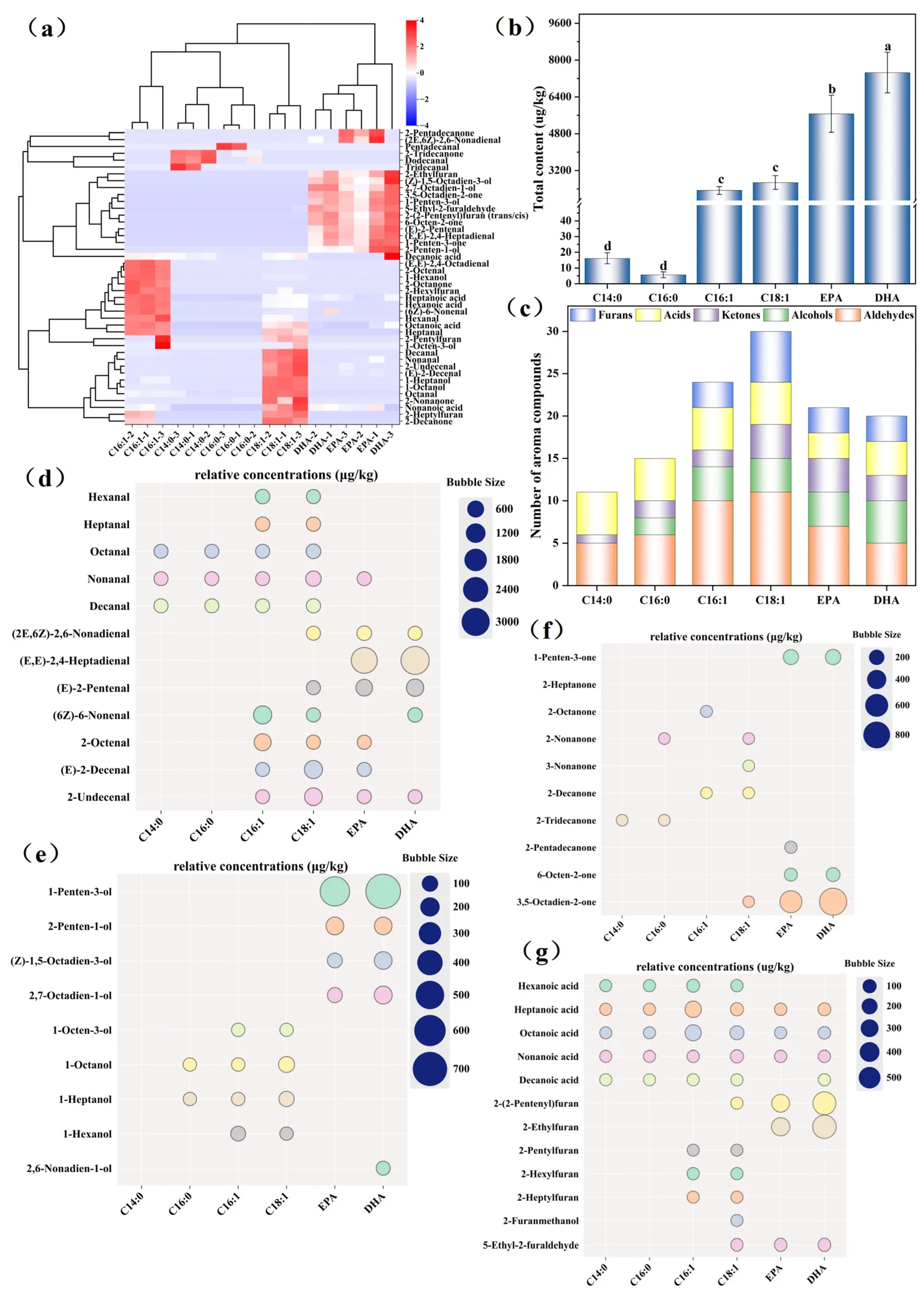
Volatile compounds generated from the thermal oxidation of six individual fatty acids. (**a**) Heatmap of volatile organic compounds (VOCs) detected in the FA-only model; (**b**) total relative VOC concentration (μg/kg) generated from each fatty acid; (**c**) number and class distribution of reproducibly detected VOCs from each fatty acid; and (**d**–**g**) relative concentrations (μg/kg) of representative aldehydes, alcohols, ketones, and furans/acids, respectively. Values are presented as mean ± SD (*n* = 3 independent preparation/heating replicates). Different lowercase letters indicate significant differences among fatty acids in panel **b** (*p* < 0.05). In panel (**a**), the color scale indicates relative VOC abundance, with red representing higher and blue representing lower values; in panel (**c**), colors distinguish VOC classes; in panels (**d**–**g**), colors distinguish individual compounds, and bubble size represents relative concentration.

The six fatty acids showed clearly different volatile profiles after heating at 115 °C for 36 min (Figure 3a). The semi-quantitative concentrations of the reproducibly detected VOCs across the 20 model-system groups are provided in Table S3. C14:0 and C16:0 produced only weak volatile responses, whereas C16:1, C18:1, EPA, and DHA showed much stronger responses. Total relative VOC concentrations were 16.14 ± 9.49 and 4.66 ± 1.89 μg/kg for C14:0 and C16:0, respectively, compared with 2331.82 ± 164.38 μg/kg for C16:1 and 2674.25 ± 496.37 μg/kg for C18:1 (Figure 3b). EPA and DHA reached 5661.31 ± 2804.55 and 7453.54 ± 3283.24 μg/kg, respectively. DHA was significantly higher than all other fatty acid treatments, whereas EPA was significantly higher than C14:0, C16:0, C16:1, and C18:1. C16:1 and C18:1 were also significantly higher than the two saturated fatty acids (*p* < 0.05). These results show that EPA and DHA gave the strongest overall volatile responses under the applied heating conditions.

The number of reproducibly detected VOCs showed a different pattern (Figure 3c). C18:1 and C16:1 produced the broadest profiles, with 30 and 24 VOCs, respectively, whereas 21 and 20 VOCs were detected in the EPA and DHA systems. C16:0 and C14:0 contained 15 and 11 VOCs, respectively. Thus, the higher total VOC concentrations of EPA and DHA did not correspond to a greater number of detected compounds. C16:1 and C18:1 produced a wider range of volatile products, whereas the EPA and DHA systems contained fewer reproducibly detected VOCs but showed substantially higher total relative VOC concentrations.

The composition of the volatile profiles also differed among the fatty acids (Figure 3d–g). C16:1 and C18:1 showed relatively broad aldehyde profiles, including hexanal, heptanal, octanal, nonanal, and unsaturated aldehydes. In the C18:1 system, nonanal and octanal reached 298.85 ± 50.23 and 152.16 ± 4.14 μg/kg, respectively, while (E)-2-decenal and 2-undecenal were also prominent. These compounds are consistent with products formed through the cleavage of oleic acid hydroperoxides [28]. EPA and DHA showed a different profile, characterized by unsaturated aldehydes, short-chain alcohols, ketones, and furan derivatives. (E,E)-2,4-Heptadienal reached 2575.72 ± 1247.33 μg/kg in the EPA system and 3029.69 ± 1549.31 μg/kg in the DHA system. Other compounds detected in these systems included (E)-2-pentenal, 1-penten-3-ol, 1-penten-3-one, 3,5-octadien-2-one, 2-ethylfuran, and 2-(2-pentenyl)furan. Similar volatile products have been reported during oxidation or heating of marine oils and EPA/DHA-containing systems [8,9,29,30].

These differences are consistent with the oxidation chemistry of the individual fatty acids. Greater unsaturation increases susceptibility to hydrogen abstraction and hydroperoxide formation, but the products formed after hydroperoxide cleavage also depend on double-bond position and hydroperoxide structure [7,25]. This helps explain why C16:1 and C18:1 produced broader volatile profiles, whereas EPA and DHA showed much higher total VOC concentrations dominated by characteristic PUFA oxidation products. The FA-only model therefore provided the precursor-level reference used to examine how these volatile patterns changed in the lipid-depleted krill and complete krill meat matrices.

### 3.4. Matrix-Dependent Changes in Volatile Profiles in the LFK + FA Model

The LFK + FA model represented the intermediate tier between the FA-only and KM + FA systems. Compared with the FA-only model, the lipid-depleted krill matrix retained krill-derived proteinaceous and other non-volatile components while reducing the contribution of endogenous lipids. This model was used to examine how the volatile profiles observed for individual fatty acids changed after incorporation into a simplified krill matrix.

**Figure 4:**
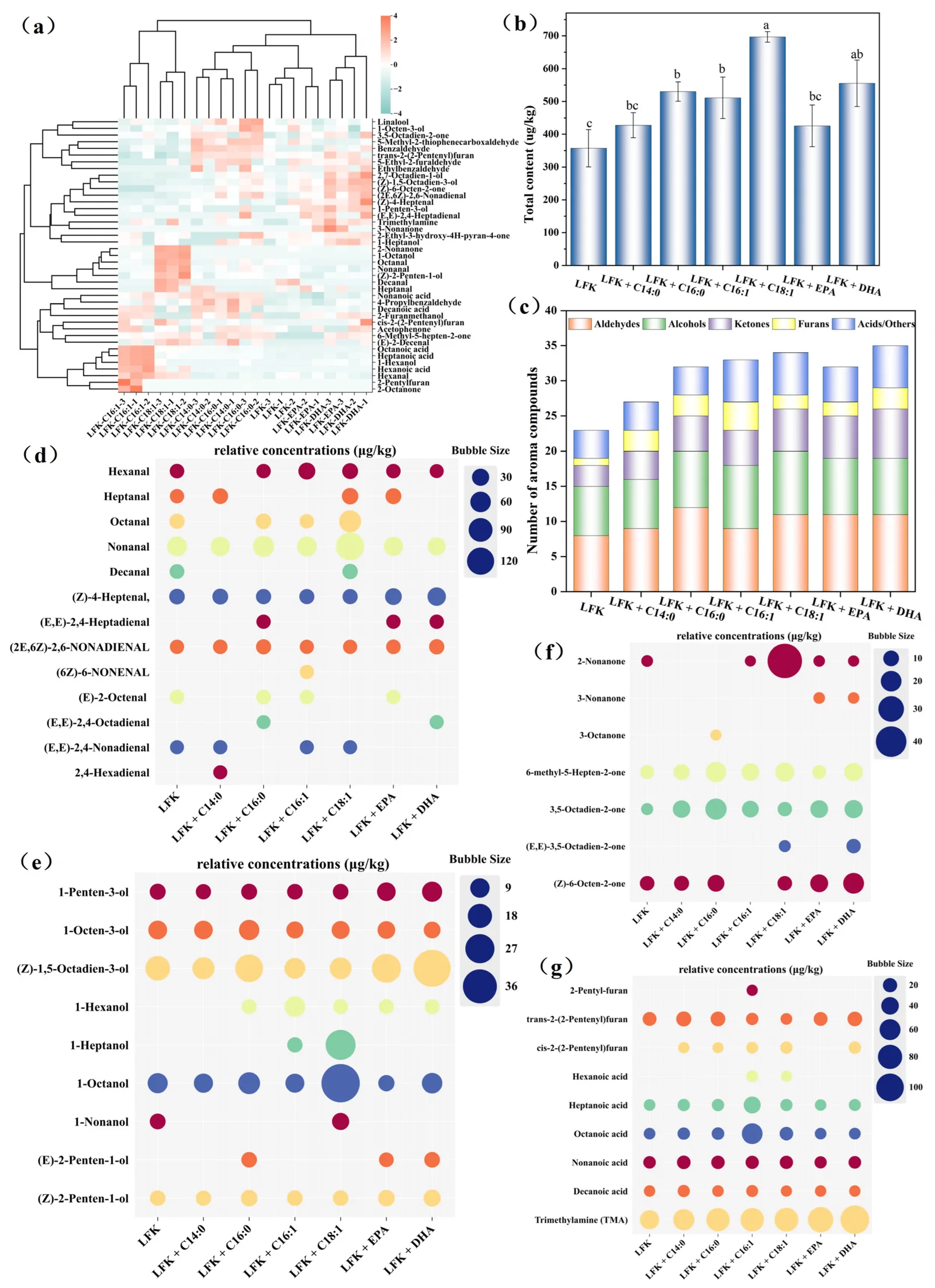
Volatile compounds tentatively identified in the lipid-depleted krill matrix (sample code LFK) and LFK + fatty acid (FA) systems. (**a**) Heatmap of VOCs detected in the LFK and LFK + FA systems; (**b**) total relative VOC concentration (μg/kg) in each system; (**c**) number and class distribution of representative VOCs that were reproducibly detected; and (**d**–**g**) relative concentrations (μg/kg) of representative aldehydes, alcohols, ketones, and furans/acids, respectively. Representative VOCs were selected on the basis of reproducible detection and relevance to lipid oxidation and/or odor expression. Values are presented as mean ± SD (*n* = 3 independent preparation/heating replicates). Different lowercase letters indicate significant differences among treatments in panel (**b**) (*p* < 0.05). In panel (**a**), the color scale indicates relative VOC abundance, with red representing higher and blue representing lower values; in panel (**c**), colors distinguish VOC classes; in panels (**d**–**g**), colors distinguish individual compounds, and bubble size represents relative concentration.

The volatile profiles of the LFK + FA systems differed clearly from that of the LFK blank and did not follow the response order observed in the FA-only model (Figure 4a). The LFK blank itself showed a measurable volatile background, indicating that the solvent-extracted matrix still contained components capable of contributing to volatile formation during heating. Total relative VOC concentrations also differed among treatments (Figure 4b). The LFK blank showed the lowest value (357.25 ± 56.77 μg/kg), whereas LFK + C18:1 showed the highest value (696.86 ± 16.13 μg/kg). LFK + DHA, LFK + C16:0, and LFK + C16:1 reached 555.21 ± 70.98, 529.99 ± 29.60, and 511.04 ± 63.13 μg/kg, respectively, while LFK + C14:0 and LFK + EPA reached 427.55 ± 38.17 and 425.41 ± 63.41 μg/kg, respectively. LFK + C18:1 was significantly higher than all groups except LFK + DHA, and LFK + DHA was significantly higher than the LFK blank (*p* < 0.05). In the FA-only model, EPA and DHA showed the highest total VOC concentrations, whereas the highest response in the LFK model occurred after C18:1 supplementation. This change in response order indicates that the volatile behavior observed for the isolated fatty acids was altered after incorporation into the krill matrix.

The number and class distribution of reproducibly detected VOCs showed a similar change (Figure 4c). The LFK blank contained 23 representative VOCs, whereas 27, 32, 33, 34, 32, and 35 VOCs were detected in the LFK + C14:0, LFK + C16:0, LFK + C16:1, LFK + C18:1, LFK + EPA, and LFK + DHA systems, respectively. LFK + DHA contained the largest number of representative VOCs, followed by LFK + C18:1 and LFK + C16:1. Fatty acid supplementation therefore changed not only the total VOC concentration but also the range of volatile compounds detected in the lipid-depleted matrix.

The individual compounds further illustrated these differences (Figure 4d–g). LFK + C18:1 showed relatively high levels of medium-chain aldehydes, including nonanal, octanal, heptanal, and decanal. Nonanal and octanal reached 128.03 ± 14.71 and 73.07 ± 4.53 μg/kg, respectively, and relatively high levels of 1-octanol, 1-heptanol, and 2-nonanone were also observed. The aldehydic profile observed after C18:1 supplementation is consistent with known products of oleic acid oxidation, which can form aldehydes, alcohols, ketones, acids, and furans through hydroperoxide- and radical-mediated pathways [28].

LFK + EPA and LFK + DHA showed a different pattern. Rather than a general increase in aldehydes, selected unsaturated oxidation products were more prominent in these treatments. For example, (Z)-4-heptenal reached 25.70 ± 4.39 μg/kg in LFK + EPA and 41.03 ± 18.87 μg/kg in LFK + DHA. LFK + DHA also showed relatively high levels of (2E,6Z)-2,6-nonadienal, 1-penten-3-ol, (Z)-1,5-octadien-3-ol, 3,5-octadien-2-one, (E,E)-3,5-octadien-2-one, (Z)-6-octen-2-one, and 2-(2-pentenyl)furan isomers. Similar compounds have been reported during oxidation of DHA-rich marine lipids and other EPA/DHA-containing systems [30]. Thus, EPA and DHA supplementation remained associated with characteristic PUFA oxidation products in the LFK matrix, although their relative expression differed from that observed in the FA-only model.

Other volatile classes also varied among the LFK + FA treatments. LFK + C16:1 showed relatively high levels of heptanoic and octanoic acids, while the two 2-(2-pentenyl)furan isomers varied among treatments. Trimethylamine remained detectable in the LFK blank at 49.60 ± 10.59 μg/kg, showing that the lipid-depleted matrix retained a nitrogen-containing volatile background after solvent extraction. These differences may reflect changes in volatile formation as well as matrix effects on partitioning and headspace release. Proteins and other non-volatile components can influence volatile binding, partitioning, and release and may also react with lipid oxidation products [11,31,32,33,34,35]. The present headspace measurements, however, do not allow these processes to be distinguished quantitatively.

Overall, the volatile patterns observed in the FA-only model changed markedly after incorporation into the lipid-depleted krill matrix. The C18:1-supplemented system showed the highest total VOC concentration together with a prominent aldehydic profile, whereas EPA- and DHA-supplemented systems showed more selective increases in unsaturated aldehydes, short-chain alcohols, ketones, and furan derivatives. These results show that the volatile expression associated with fatty acid supplementation was strongly affected by the surrounding krill matrix.

### 3.5. Fatty Acid-Associated Volatile Profiles in the Complete Krill Matrix

The KM + FA model was the final tier of the three-tier thermal oxidation model system. Unlike the FA-only and lipid-depleted krill models, complete krill meat contained endogenous lipids, proteins, nitrogen-containing precursors, and other native matrix constituents. This tier was used to examine how the volatile patterns observed in the preceding models changed in the complete krill matrix.

**Figure 5:**
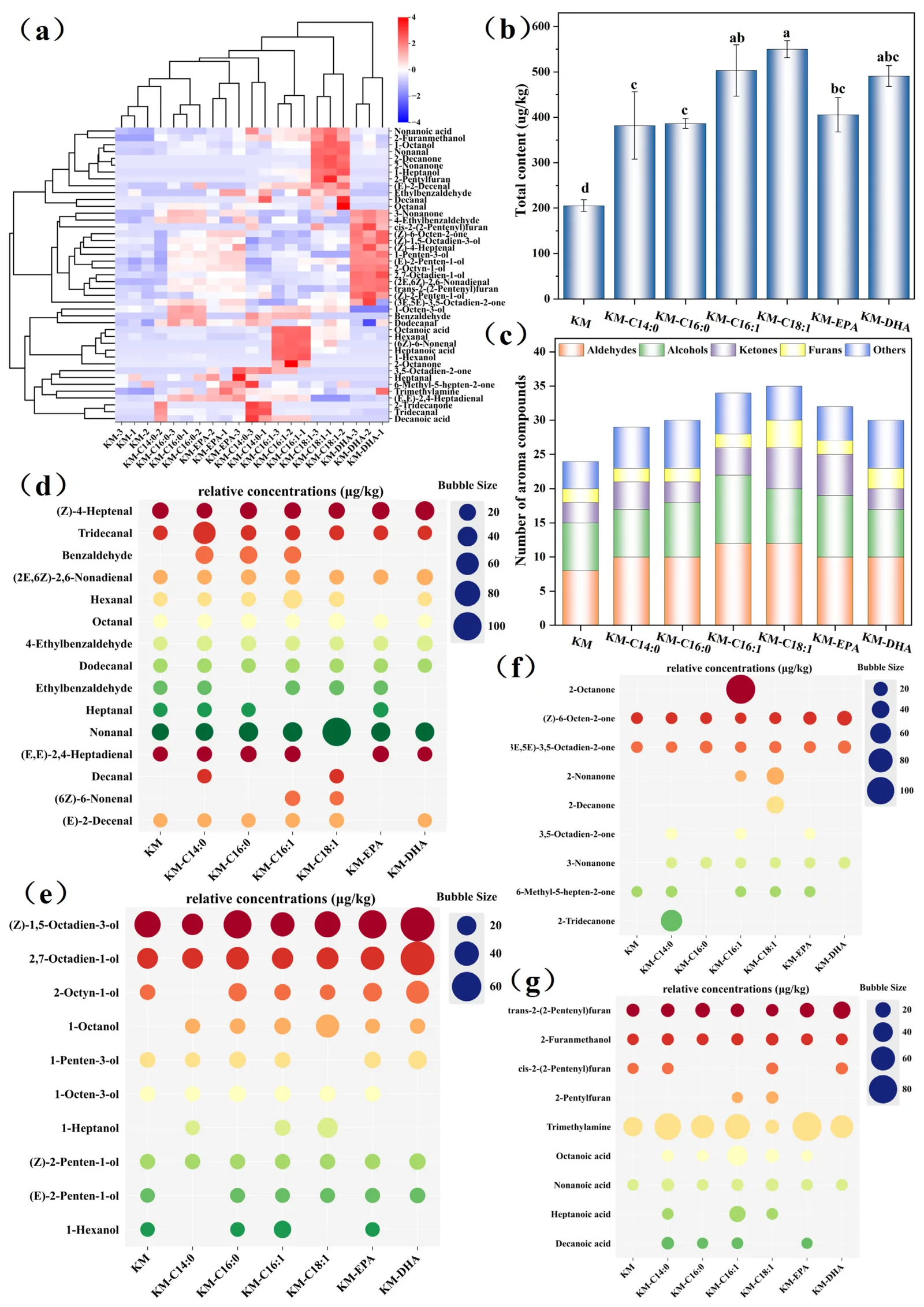
Volatile compounds tentatively identified in the krill meat (KM) and KM + fatty acid (FA) systems. (**a**) Heatmap of VOCs detected in the KM and KM + FA systems; (**b**) total relative VOC concentration (μg/kg) in each system; (**c**) number and class distribution of representative VOCs that were reproducibly detected; and (**d**–**g**) relative concentrations (μg/kg) of representative aldehydes, alcohols, ketones, and furans/other compounds, respectively. Representative VOCs were selected on the basis of reproducible detection and relevance to lipid oxidation and/or odor expression. Values are presented as mean ± SD (*n* = 3 independent preparation/heating replicates). Different lowercase letters indicate significant differences among treatments in panel **b** (*p* < 0.05). In panel (**a**), the color scale indicates relative VOC abundance, with red representing higher and blue representing lower values; in panel (**c**), colors distinguish VOC classes; in panels (**d**–**g**), colors distinguish individual compounds, and bubble size represents relative concentration.

The KM + FA systems showed distinct volatile profiles from the KM blank (Figure 5a). The KM blank already showed a complex volatile background, consistent with the presence of endogenous lipid and non-lipid precursors in krill meat. Fatty acid supplementation further changed this profile, but the response pattern differed from both the FA-only and LFK + FA models. Total relative VOC concentrations increased significantly after fatty acid supplementation (Figure 5b). The KM blank showed the lowest value at 205.51 ± 12.98 μg/kg, whereas KM + C18:1 showed the highest value at 549.96 ± 18.63 μg/kg. KM + C16:1 and KM + DHA also showed high total VOC concentrations of 503.07 ± 56.66 and 490.69 ± 23.00 μg/kg, respectively. KM + EPA, KM + C16:0, and KM + C14:0 reached 405.67 ± 37.92, 386.45 ± 10.79, and 381.87 ± 74.12 μg/kg, respectively. All fatty-acid-supplemented groups were significantly higher than the KM blank (*p* < 0.05). KM + C18:1 was significantly higher than KM + EPA, KM + C16:0, and KM + C14:0, but did not differ significantly from KM + C16:1 or KM + DHA. Thus, unlike the more distinct C18:1 response observed in the LFK model, the complete krill matrix showed similarly high responses to C18:1, C16:1, and DHA supplementation.

The number and class distribution of reproducibly detected VOCs also differed among treatments (Figure 5c). The KM blank contained 24 representative VOCs, whereas 29–35 VOCs were detected in the fatty-acid-supplemented systems. KM + C18:1 contained the largest number of representative VOCs, followed by KM + C16:1 and KM + EPA. Aldehydes remained an important class, while alcohols, ketones, furans, acids, and other compounds contributed additional differences among treatments. These results show that fatty acid supplementation changed both the abundance and composition of volatiles against the existing KM background.

The aldehyde profiles showed clear treatment-related differences (Figure 5d). KM + C18:1 was characterized by a marked increase in nonanal, which reached 101.81 ± 2.56 μg/kg, together with relatively high levels of octanal, (E)-2-decenal, and decanal. The prominence of these medium-chain aldehydes is consistent with the known oxidation chemistry of oleic acid, in which hydroperoxide- and radical-mediated reactions can form aldehydes and other carbonyl compounds [28]. In contrast, KM + DHA showed the highest level of (Z)-4-heptenal at 36.15 ± 6.01 μg/kg and a relatively high level of (2E,6Z)-2,6-nonadienal at 15.35 ± 0.22 μg/kg. KM + EPA also contained relatively high levels of (Z)-4-heptenal (25.59 ± 6.85 μg/kg) and (E,E)-2,4-heptadienal (17.16 ± 1.47 μg/kg). These compounds are commonly associated with the oxidation of long-chain marine n-3 PUFAs [30].

Alcohols and ketones showed further differences among the KM + FA treatments (Figure 5e,f). KM + DHA contained relatively high levels of (Z)-1,5-octadien-3-ol, 2,7-octadien-1-ol, 2-octyn-1-ol, and 1-penten-3-ol. KM + C16:1 was characterized by a marked increase in 2-octanone, whereas KM + C18:1 showed relatively high levels of 2-nonanone and 2-decanone. KM + DHA also showed high levels of (Z)-6-octen-2-one and (3E,5E)-3,5-octadien-2-one. These differences show that the unsaturated-fatty-acid treatments did not produce a uniform volatile pattern in the complete krill matrix.

Furans, organic acids, and nitrogen-containing compounds added further differences to the KM volatile profile (Figure 5g). The two 2-(2-pentenyl)furan isomers varied among treatments, whereas KM + C16:1 showed relatively high levels of heptanoic and octanoic acids. Trimethylamine was already present in the KM blank at 33.91 ± 3.92 μg/kg and varied after fatty acid supplementation, reaching 92.39 ± 13.79 μg/kg in KM + EPA. The presence of TMA in the untreated KM background is particularly relevant because it shows that the complete krill matrix contained nitrogen-containing volatiles in addition to lipid-oxidation-related compounds. Proteins, endogenous lipids, nitrogen-containing precursors, and other matrix components may also affect volatile formation, partitioning, and headspace release during heating [11,31,32,33,34,35,36].

Overall, the complete krill matrix did not simply reproduce the volatile patterns observed in the FA-only or LFK + FA systems. C18:1 supplementation was associated mainly with a pronounced medium-chain aldehyde profile, C16:1 supplementation with distinct ketone and organic-acid changes, and EPA/DHA supplementation with unsaturated aldehydes, alcohols, ketones, and furans commonly associated with marine PUFA oxidation. At the same time, TMA and other endogenous volatiles contributed to the KM background. The volatile profile expressed in complete krill meat was therefore shaped by both fatty acid supplementation and the native matrix environment.

### 3.6. Odor Relevance of Fatty Acid-Associated Oxidation Products in the Three Model Systems

**Figure 6:**
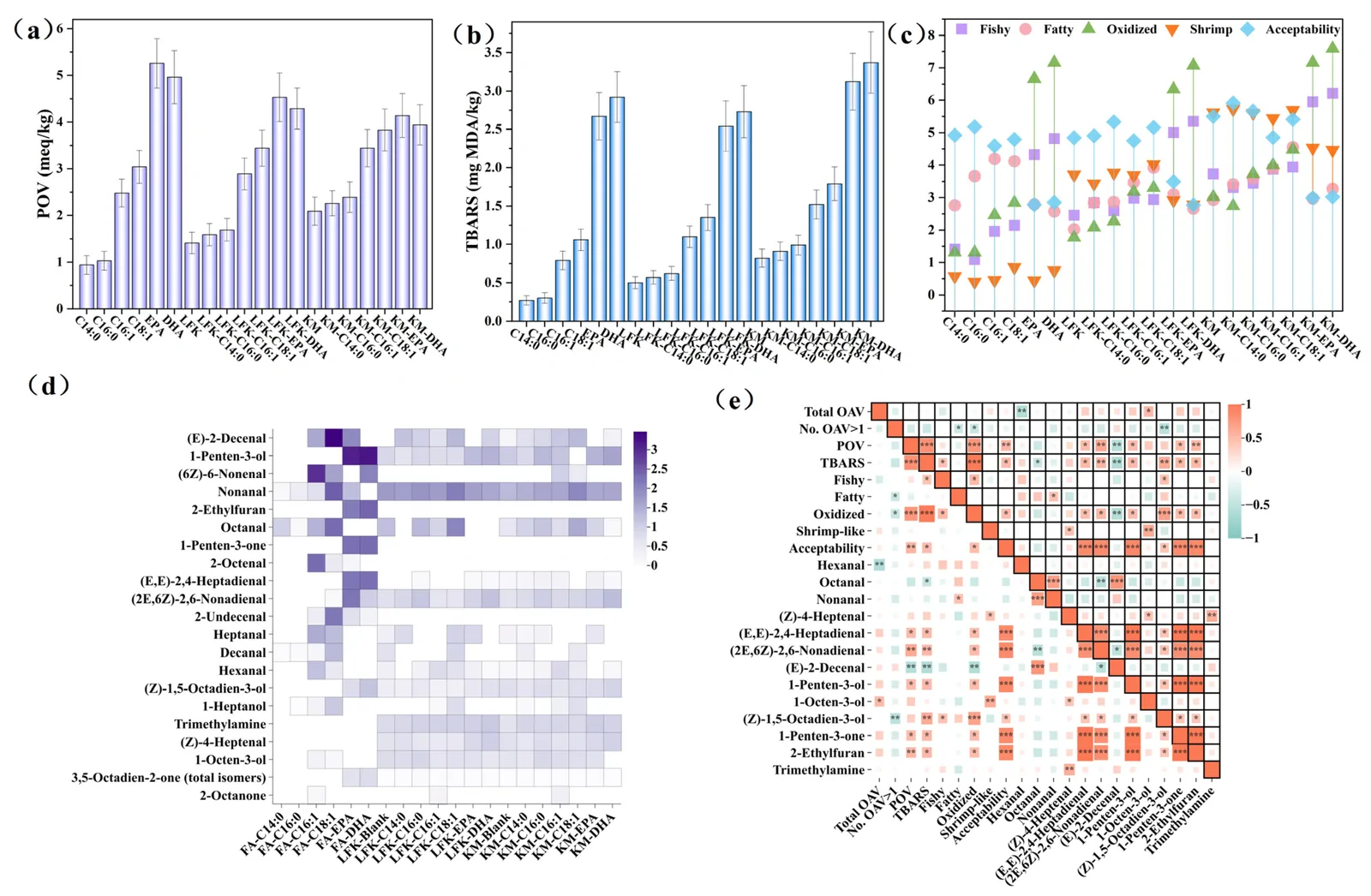
Oxidation status, potentially odor-active compounds, sensory responses, and exploratory correlations across the three model systems. (**a**) Peroxide value (POV) of the FA-only, LFK + FA, and KM + FA systems; (**b**) thiobarbituric acid-reactive substances (TBARS) values; (**c**) fishy, fatty, oxidized, and shrimp-like odor intensities and overall odor acceptability; (**d**) heatmap of compounds with OAV > 1 across all 20 groups, displayed as log10(OAV + 1) for visualization; and (**e**) exploratory Pearson correlation heatmap based on the treatment means of all 20 groups, including oxidation indices, sensory attributes, total OAV, the number of compounds with OAV > 1, and selected potentially odor-active compounds. Absolute total OAV magnitudes were not directly ranked among the FA-only, LFK + FA, and KM + FA models. Panels (**a**,**b**) are presented as mean ± SD from three independent preparation/heating replicates, and panel (**c**) summarizes sensory scores from 30 trained panelists. In panel (**e**), *, **, and *** indicate *p* < 0.05, *p* < 0.01, and *p* < 0.001, respectively.

Oxidation indices, potentially odor-active compounds, sensory responses, and correlation patterns were examined together to evaluate odor deterioration across the FA-only, LFK + FA, and KM + FA systems (Figure 6). Pearson correlation analysis was based on the treatment means of all 20 groups and was used as an exploratory cross-tier assessment. Because the three model tiers differed in composition, headspace partitioning, and VOC normalization basis, the pooled correlations were interpreted as associations across the full dataset rather than as evidence of causal relationships within an individual model system.

POV and TBARS showed different responses to fatty acid type and matrix environment (Figure 6a,b). FA-EPA and FA-DHA had the highest POVs, reaching 5.26 ± 0.53 and 4.96 ± 0.57 meq/kg, respectively, indicating the high primary oxidation susceptibility of these long-chain n-3 PUFAs under the applied heating conditions. TBARS showed a stronger response in the complete krill matrix, with KM + DHA and KM + EPA reaching the highest values of 3.37 ± 0.40 and 3.12 ± 0.37 mg MDA/kg, respectively. Thus, EPA and DHA showed pronounced primary oxidation in the FA-only model, whereas high secondary oxidation levels were observed in the corresponding EPA- and DHA-supplemented KM systems. This pattern was consistent with the volatile profiles of the KM + FA model, in which these treatments contained relatively high levels of unsaturated aldehydes, alcohols, ketones, and furan derivatives commonly associated with marine PUFA oxidation [8,9,29,30].

Sensory evaluation also differed markedly among treatments (Figure 6c). Linear mixed-effects analysis confirmed a significant overall treatment effect for all five sensory attributes (all *p* < 0.001). EPA- and DHA-containing systems generally showed stronger fishy and oxidized odors, particularly in the krill-derived matrices. KM + DHA and KM + EPA had the highest fishy scores, reaching 6.21 and 5.95, respectively, and highly oxidized EPA- and DHA-containing treatments also tended to show lower odor acceptability. The high oxidation levels observed in these systems were therefore accompanied by less desirable odor profiles, although the extent of the sensory changes differed among the three model tiers.

OAV analysis showed that the potentially odor-active profiles also changed among the three model tiers (Figure 6d), with the corresponding odor thresholds and calculated OAVs provided in Table S4. Because VOC concentrations were obtained using single-internal-standard semi-quantification, compounds with OAV > 1 were considered potentially odor-active compounds rather than quantitative measures of absolute sensory contributions. In the FA-only model, C16:1 and C18:1 were characterized mainly by aldehydic compounds, whereas EPA and DHA showed high OAVs for compounds including 1-penten-3-ol, 1-penten-3-one, (E,E)-2,4-heptadienal, (2E,6Z)-2,6-nonadienal, and 2-ethylfuran. After incorporation into the LFK and KM matrices, these profiles changed substantially. Nonanal remained prominent in several matrix-containing treatments, while (Z)-4-heptenal, (2E,6Z)-2,6-nonadienal, 1-penten-3-ol, 1-octen-3-ol, (Z)-1,5-octadien-3-ol, and TMA varied among treatments. Absolute total OAV values were not directly ranked among the FA-only, LFK + FA, and KM + FA models because of differences in model composition, headspace behavior, and concentration basis.

The odor characteristics of individual compounds help explain why a high total OAV did not necessarily correspond to the strongest fishy or oxidized odor. Octanal and nonanal are generally associated with oily, fatty, green, or citrus-like notes, whereas (E)-2-decenal is more closely related to fatty, waxy, and oxidized-lipid odors [16,20]. Their prominence in C18:1-containing systems therefore corresponds more closely to an aldehydic and fatty odor profile than to the strongest fishy response. In contrast, several compounds that were prominent in EPA/DHA-containing and krill-matrix systems have odor characteristics more closely related to marine, fishy, green, or oxidized notes.

Exploratory Pearson correlation analysis across the 20 treatment means provided further information on these relationships (Figure 6e). POV was positively correlated with fishy odor (r = 0.794, *p* < 0.001) and oxidized odor (r = 0.912, *p* < 0.001) and negatively correlated with odor acceptability (r = −0.751, *p* < 0.001). TBARS showed even stronger associations with fishy odor (r = 0.926, *p* < 0.001) and oxidized odor (r = 0.991, *p* < 0.001) and was negatively correlated with acceptability (r = −0.859, *p* < 0.001). These pooled associations are consistent with greater oxidation being accompanied by poorer odor quality across the full model dataset, but they do not establish causality within an individual model tier.

Individual compounds showed different associations with the sensory attributes. (Z)-4-Heptenal was positively correlated with fishy odor (r = 0.707, *p* < 0.001) and oxidized odor (r = 0.520, *p* = 0.020). (Z)-1,5-Octadien-3-ol was positively correlated with fishy odor (r = 0.702, *p* < 0.001) and oxidized odor (r = 0.720, *p* < 0.001) and negatively correlated with odor acceptability (r = −0.520, *p* = 0.019). TMA was positively correlated with fishy odor (r = 0.501, *p* = 0.024), but not significantly with oxidized odor or acceptability. In contrast, 1-penten-3-ol was not significantly correlated with fishy odor (r = 0.275, *p* = 0.240), but was positively correlated with oxidized odor (r = 0.470, *p* = 0.035) and negatively correlated with acceptability (r = −0.540, *p* = 0.014). The fishy and oxidized attributes were therefore associated with partly different compounds: (Z)-4-heptenal and TMA were more closely related to fishy odor, 1-penten-3-ol to oxidized odor, and (Z)-1,5-octadien-3-ol to both attributes.

Total OAV, however, was not significantly correlated with fishy odor (r = −0.051, *p* = 0.831) or oxidized odor (r = 0.181, *p* = 0.445). Sensory deterioration across the three model tiers therefore could not be explained by summed OAV alone. The identity and odor characteristics of individual compounds, together with changes in their expression among the different matrices, were more informative for distinguishing fishy and oxidized odor patterns. Because the correlations were calculated from pooled treatment means, these relationships should be interpreted at the cross-tier level rather than as direct effects within a particular matrix.

**Figure 7:**
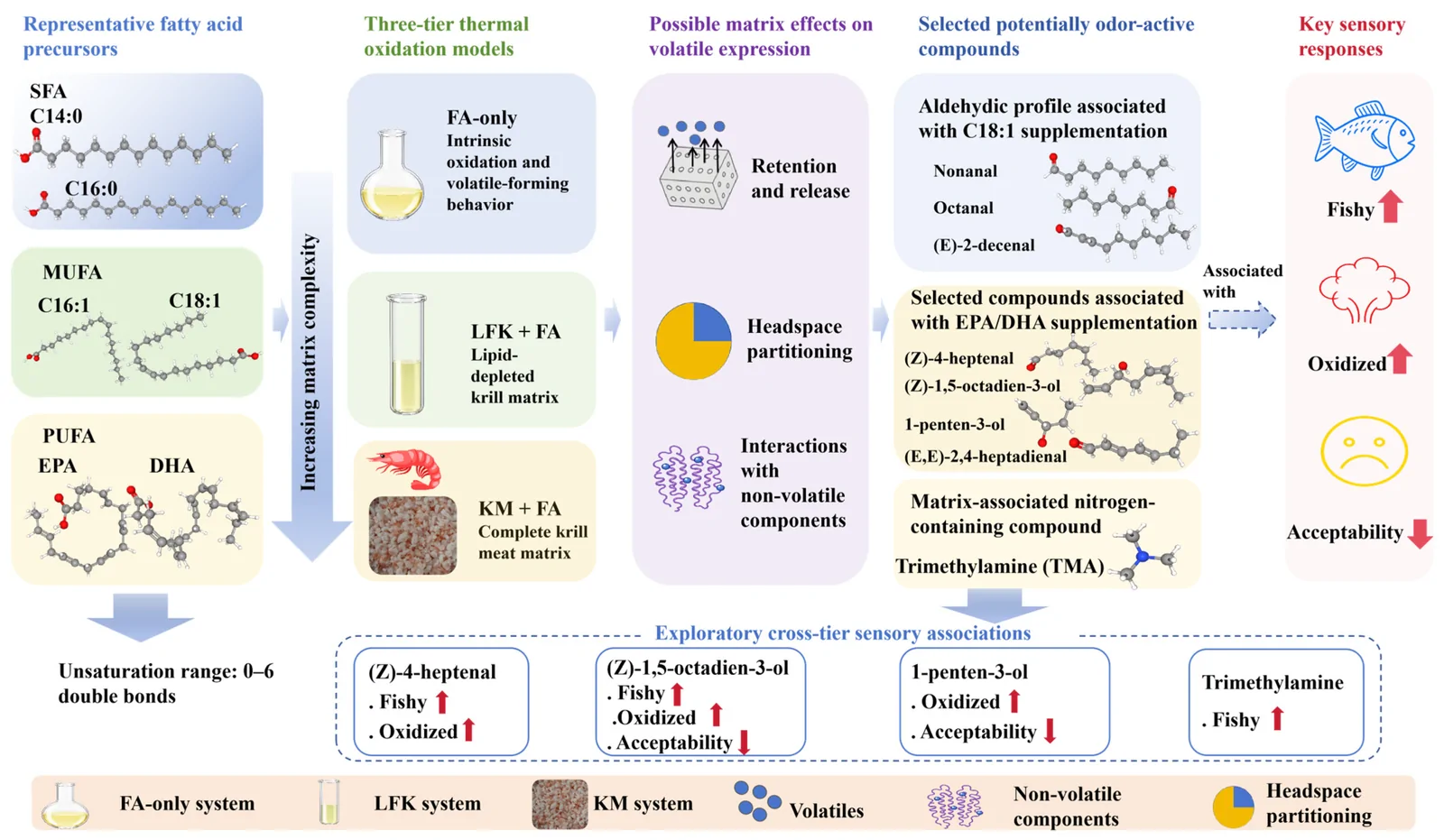
Integrated schematic of fatty acid oxidation and matrix-dependent volatile expression in the three-tier thermal oxidation model system. The FA-only tier represents the intrinsic oxidation and volatile-forming behavior of individual fatty acids, whereas the LFK + FA and KM + FA tiers illustrate changes in volatile expression with increasing matrix complexity. Compounds shown were selected from the OAV-screened profiles and are therefore described as potentially odor-active compounds. Sensory links at the bottom represent exploratory cross-tier associations based on the treatment means of all 20 groups and do not imply causal relationships within individual model systems. Red upward and downward arrows indicate higher and lower sensory responses, respectively. The blue/yellow circular symbol schematically represents headspace partitioning; its color proportions are illustrative and do not represent quantitative values.

Figure 6 and Figure 7 together summarize the progression from fatty acid oxidation to odor expression in the three-tier model system. C18:1-containing systems were characterized mainly by aldehydic compounds, whereas EPA- and DHA-containing systems showed stronger primary or secondary oxidation and greater prominence of several unsaturated aldehydes and alcohols associated with fishy and oxidized odors. These patterns changed further after incorporation into krill matrices and were accompanied by matrix-associated volatiles such as TMA. Taken across the three model tiers, off-odor deterioration was more closely associated with the matrix-dependent expression of selected potentially odor-active compounds than with fatty acid unsaturation, total VOC concentration, or total OAV alone.

Several limitations should be considered when interpreting these results. VOC concentrations were estimated by single-internal-standard semi-quantification, and post-heating residual fatty acid concentrations were not determined; accordingly, OAVs were treated as comparative indicators of potential odor relevance, and precursor depletion was not quantified directly. Because the three model tiers differed in concentration normalization and headspace behavior, absolute VOC and OAV magnitudes were not compared across tiers, and pooled correlations were interpreted as exploratory rather than causal. Finally, odor acceptability reflects trained-panel ratings rather than consumer liking, and the individual-fatty-acid models do not reproduce the complete lipid composition of commercial Antarctic krill products.

## 4. Conclusions

The three-tier thermal oxidation model system distinguished the intrinsic oxidation and volatile-forming behavior of representative Antarctic krill fatty acids from their odor expression in krill-derived matrices. In the FA-only model, EPA and DHA showed the strongest primary oxidation, with POVs of 5.26 ± 0.53 and 4.96 ± 0.57 meq/kg, respectively, whereas C16:1 and C18:1 produced broader volatile profiles. These patterns changed after incorporation into the krill matrices. C18:1 supplementation was associated with a pronounced aldehydic profile, while the EPA- and DHA-supplemented complete krill systems showed the highest secondary oxidation levels, with TBARS values of 3.12 ± 0.37 and 3.37 ± 0.40 mg MDA/kg, respectively, and high fishy odor scores of 5.95 and 6.21.

Off-odor deterioration was not adequately described by fatty acid unsaturation, total VOC concentration, or total OAV alone. Instead, the odor response was more closely related to the matrix-dependent expression of selected potentially odor-active compounds. (Z)-4-Heptenal and (Z)-1,5-octadien-3-ol were associated with both fishy and oxidized odors, 1-penten-3-ol was more closely associated with oxidized odor and reduced acceptability, and TMA showed a specific association with fishy odor. These results show that the odor impact of fatty acid oxidation is closely related to how individual volatile compounds are expressed within the krill matrix. The three-tier model also helps identify oxidation-related compounds that may be targeted in future strategies to reduce off-odor formation during thermal processing of Antarctic krill. Further validation of these compounds by GC–olfactometry and aroma recombination or omission experiments in commercial krill products is warranted.

## Data Availability

The original contributions presented in this study are included in the article/Supplementary Materials. Further inquiries can be directed to the corresponding author.

## Supplementary Materials

The following supporting information can be downloaded at https://www.mdpi.com/article/10.3390/foods15173053/s1. Table S1: Sample codes, model composition, and descriptions of the 20 thermal oxidation model groups; Table S2: Sensory attributes, definitions, and reference materials used for odor evaluation; Table S3: Semi-quantitative concentrations of volatile compounds reproducibly detected across the 20 model-system groups; Table S4: Odor thresholds and odor activity values of volatile compounds across the 20 thermal oxidation model groups. Threshold references used in Table S4 are provided in the Supplementary Materials.
