# Supplementary material for: From Fatty Acid Precursors to Off-Odor in Canned Antarctic Krill: Matrix-Dependent Odorant Expression Under Retort Sterilization Conditions
Source: Foods. 2026 Aug 28;15(17):3053. doi: 10.3390/foods15173053 (PMC13564515; doi:10.3390/foods15173053)
Supplement: Supplementary file 1 [file foods-15-03053-s001.zip › foods-4482691-supplementary.pdf]

Supplementary Materials

From Fatty Acid Precursors to Off-Odor in Canned Antarctic Krill: Matrix-Dependent Odorant Expression Under Retort Sterilization Conditions

Peizi Sun[1], Songyi Lin[1,2,3,4], Yuting Tan[1], Yajie Qin[1], Xiaobin Bi[1] and Dongmei Li[1,2,3,4,*]

[1] School of Food Science and Technology, National Engineering Research Center of Seafood, Dalian Polytechnic University, Dalian 116034, China

[2] Engineering Research Center of Seafood of Ministry of Education of China, Dalian 116034, China

[3] Collaborative Innovation Center of Seafood Deep Processing, Dalian Polytechnic University, Dalian 116034, China

[4] State Key Laboratory of Marine Food Processing and Safety Control, Dalian Polytechnic University, Dalian 116034, China

*** Correspondence:** lidongmei@dlpu.edu.cn; Tel./Fax: +86-411-86323262; ORCID: 0000-0002-5961-4176

Table S1. Sample codes, model composition, and descriptions of the 20 thermal oxidation model groups.

Group code	Fatty acid / matrix	Model system	Description
FA1	C14:0 / myristic acid	M1: FA-only	Myristic acid-only heated model
FA2	C16:0 / palmitic acid	M1: FA-only	Palmitic acid-only heated model
FA3	C16:1 / palmitoleic acid	M1: FA-only	Palmitoleic acid-only heated model
FA4	C18:1 / oleic acid	M1: FA-only	Oleic acid-only heated model
FA5	EPA / eicosapentaenoic acid	M1: FA-only	EPA-only heated model
FA6	DHA / docosahexaenoic acid	M1: FA-only	DHA-only heated model
LFK	LFK matrix	M2: LFK	Lipid-depleted krill matrix without fatty acid addition
LFK-FA1	LFK + C14:0	M2: LFK + FA	LFK supplemented with myristic acid
LFK-FA2	LFK + C16:0	M2: LFK + FA	LFK supplemented with palmitic acid
LFK-FA3	LFK + C16:1	M2: LFK + FA	LFK supplemented with palmitoleic acid
LFK-FA4	LFK + C18:1	M2: LFK + FA	LFK supplemented with oleic acid
LFK-FA5	LFK + EPA	M2: LFK + FA	LFK supplemented with EPA
LFK-FA6	LFK + DHA	M2: LFK + FA	LFK supplemented with DHA
KM	KM matrix	M3: KM	Krill meat matrix without fatty acid addition
KM-FA1	KM + C14:0	M3: KM + FA	Krill meat matrix supplemented with myristic acid
KM-FA2	KM + C16:0	M3: KM + FA	Krill meat matrix supplemented with palmitic acid
KM-FA3	KM + C16:1	M3: KM + FA	Krill meat matrix supplemented with palmitoleic acid
KM-FA4	KM + C18:1	M3: KM + FA	Krill meat matrix supplemented with oleic acid
KM-FA5	KM + EPA	M3: KM + FA	Krill meat matrix supplemented with EPA
KM-FA6	KM + DHA	M3: KM + FA	Krill meat matrix supplemented with DHA

Note: LFK is the sample code for the lipid-depleted krill matrix; KM, krill meat matrix; FA-only, fatty-acid-only model.

Table S2. Sensory attributes, definitions, and reference materials used for odor evaluation.

Attribute	Definition	Reference material used for panel training
Fishy	Characteristic fishy odor associated with aquatic products.	Trimethylamine-like odor solution or fresh aquatic-product odor reference.
Fatty	Mild oily, fatty, or heated lipid-like aroma.	Heated edible oil or heated fatty-acid model reference.
Oxidized	Stale, oxidized oil-like, or irritating odor associated with lipid oxidation.	Oxidized oil or thermally oxidized lipid reference.
Shrimp-like	Characteristic shrimp-like aroma of krill or shrimp.	Cooked shrimp or heated krill reference.
Acceptability	Overall acceptability of the sample odor.	Overall odor impression of the tested sample.

Note: The first four attributes were evaluated as odor-intensity attributes, whereas acceptability was evaluated separately as a panel-rated overall odor acceptability response rather than consumer liking. Reference materials were used only for panel training and attribute familiarization.

Table S3. Semi-quantitative concentrations of volatile compounds reproducibly detected across the 20 model-system groups.

VOC information			FA-only model						LFK + FA model							KM + FA model						
No.	Chemical class	Compound	C14:0	C16:0	C16:1	C18:1	EPA	DHA	LFK	LFK+C14:0	LFK+C16:0	LFK+C16:1	LFK+C18:1	LFK+EPA	LFK+DHA	KM	KM+C14:0	KM+C16:0	KM+C16:1	KM+C18:1	KM+EPA	KM+DHA
1	Aldehyde	Hexanal	ND	ND	79.56 ± 14.62	12.45 ± 7.87	ND	ND	6.06 ± 5.32	ND	4.73 ± 1.14	27.92 ± 5.70	18.15 ± 1.96	5.34 ± 3.08	1.34 ± 1.36	2.10 ± 1.56	1.83 ± 2.10	3.71 ± 0.20	32.05 ± 1.38	9.80 ± 0.98	ND	1.54 ± 1.77
2	Aldehyde	Heptanal	ND	ND	87.98 ± 5.06	53.70 ± 7.68	ND	ND	3.80 ± 6.59	16.05 ± 13.94	ND	ND	22.94 ± 20.03	17.70 ± 7.96	ND	2.29 ± 2.63	1.51 ± 2.61	2.65 ± 3.04	ND	ND	8.71 ± 8.64	ND
3	Aldehyde	Octanal	6.31 ± 7.70	0.15 ± 0.13	17.02 ± 2.91	152.16 ± 4.14	ND	ND	12.15 ± 5.15	ND	14.90 ± 12.95	5.48 ± 4.77	73.07 ± 4.53	ND	ND	2.16 ± 1.89	5.68 ± 0.83	9.72 ± 0.22	3.68 ± 1.54	23.31 ± 26.71	4.91 ± 4.64	0.19 ± 0.33
4	Aldehyde	Nonanal	0.39 ± 0.10	1.86 ± 1.89	4.55 ± 0.62	298.85 ± 50.23	18.22 ± 25.96	ND	52.97 ± 8.29	47.44 ± 3.57	65.28 ± 8.11	51.92 ± 0.86	128.03 ± 14.71	47.22 ± 10.37	37.07 ± 8.06	21.04 ± 1.48	28.31 ± 2.70	37.12 ± 0.58	36.98 ± 1.47	101.81 ± 2.56	35.76 ± 6.14	36.23 ± 3.13
5	Aldehyde	Decanal	0.18 ± 0.18	0.33 ± 0.05	1.35 ± 0.41	41.84 ± 5.39	ND	ND	8.07 ± 7.21	ND	ND	ND	14.14 ± 3.45	ND	ND	ND	3.73 ± 4.27	ND	ND	4.68 ± 5.36	ND	ND
6	Aldehyde	Dodecanal	0.11 ± 0.02	0.01 ± 0.02	ND	ND	ND	ND	ND	ND	ND	ND	ND	ND	ND	1.77 ± 0.45	3.61 ± 0.36	3.94 ± 0.55	2.42 ± 0.36	2.97 ± 0.58	2.96 ± 0.25	1.75 ± 1.65
7	Aldehyde	Tridecanal	2.34 ± 2.07	ND	ND	ND	ND	ND	ND	ND	ND	ND	ND	ND	ND	3.51 ± 0.16	52.32 ± 10.23	9.79 ± 0.71	7.05 ± 0.75	6.43 ± 0.59	6.65 ± 1.49	0.93 ± 1.61
8	Aldehyde	Pentadecanal	ND	0.42 ± 0.36	ND	ND	ND	ND	ND	ND	ND	ND	ND	ND	ND	ND	ND	ND	ND	ND	ND	ND

9	Aldehyde	(Z)-4-Heptenal	ND	ND	ND	ND	ND	ND	15.36 ± 15.51	17.31 ± 1.96	12.03 ± 7.83	10.57 ± 2.04	11.06 ± 4.09	25.70 ± 4.39	41.03 ± 18.87	16.13 ± 0.92	13.10 ± 2.73	21.30 ± 1.91	14.60 ± 2.82	16.94 ± 2.86	25.59 ± 6.85	36.15 ± 6.01
10	Aldehyde	(E,E)-2,4-Heptadienal	ND	ND	ND	ND	2575.72 ± 1247.33	3029.69 ± 1549.31	ND	ND	4.08 ± 4.53	ND	ND	3.69 ± 3.36	4.89 ± 4.67	6.36 ± 0.36	9.38 ± 1.69	15.61 ± 1.31	12.75 ± 1.02	ND	17.16 ± 1.47	5.62 ± 0.58
11	Aldehyde	(2E,6Z)-2,6-Nonadienal	ND	ND	ND	0.22 ± 0.38	136.83 ± 79.48	7.75 ± 13.43	3.36 ± 0.92	5.38 ± 0.24	10.86 ± 1.05	2.57 ± 0.21	2.64 ± 0.77	6.13 ± 0.35	12.29 ± 1.53	2.85 ± 0.08	4.48 ± 1.02	8.29 ± 0.70	5.01 ± 0.53	5.91 ± 0.42	7.95 ± 0.33	15.35 ± 0.22
12	Aldehyde	(6Z)-6-Nonenal	ND	ND	907.07 ± 77.65	43.34 ± 6.58	ND	120.09 ± 20.01	ND	ND	ND	ND	ND	ND	ND	ND	ND	ND	9.41 ± 0.30	2.17 ± 2.49	ND	ND
13	Aldehyde	2-Octenal	ND	ND	620.21 ± 54.98	7.19 ± 1.05	2.02 ± 3.50	ND	ND	ND	ND	ND	ND	ND	ND	ND	ND	ND	ND	ND	ND	ND
14	Aldehyde	(E,E)-2,4-Octadienal	ND	ND	5.85 ± 0.58	ND	ND	ND	ND	ND	ND	ND	ND	ND	ND	ND	ND	ND	ND	ND	ND	ND
15	Aldehyde	(E)-2-Decenal	ND	ND	11.12 ± 0.20	882.34 ± 182.69	27.55 ± 17.85	ND	ND	4.67 ± 4.70	2.84 ± 2.56	1.31 ± 2.27	6.72 ± 1.19	ND	2.55 ± 2.23	0.18 ± 0.31	1.25 ± 1.43	2.05 ± 2.35	3.87 ± 0.01	6.33 ± 0.97	ND	0.29 ± 0.50
16	Aldehyde	2-Undecenal	ND	ND	8.18 ± 0.87	786.99 ± 203.99	22.10 ± 8.27	3.90 ± 6.76	ND	ND	ND	ND	ND	ND	ND	ND	ND	ND	ND	ND	ND	ND
17	Aldehyde	(E)-2-Pentenal	ND	ND	ND	1.86 ± 3.22	559.31 ± 271.66	700.93 ± 359.27	ND	ND	ND	ND	ND	ND	ND	ND	ND	ND	ND	ND	ND	ND
18	Aldehyde	5-Ethyl-2-furaldehyde	ND	ND	ND	0.57 ± 0.98	19.78 ± 8.33	27.47 ± 5.28	2.35 ± 2.34	3.94 ± 2.31	3.74 ± 3.26	ND	1.05 ± 1.82	3.33 ± 0.43	4.26 ± 0.85	ND	ND	ND	ND	ND	ND	ND
19	Aldehyde	Benzaldehyde	ND	ND	ND	ND	ND	ND	36.04 ± 5.65	64.31 ± 5.46	61.70 ± 3.01	37.35 ± 7.74	33.10 ± 7.21	32.81 ± 5.90	50.71 ± 8.01	ND	15.31 ± 14.14	24.69 ± 0.63	21.81 ± 0.48	ND	ND	ND

20	Aldehyde	4-Propylbenzaldehyde	ND	ND	ND	ND	ND	ND	2.12 ± 0.44	5.29 ± 0.84	4.03 ± 0.23	3.07 ± 0.97	2.60 ± 0.49	2.28 ± 0.92	2.46 ± 0.48	ND	ND	ND	ND	ND	ND	ND
21	Aldehyde	Ethylbenzaldehyde	ND	ND	ND	ND	ND	ND	2.22 ± 3.85	5.28 ± 9.14	9.31 ± 5.83	ND	ND	2.40 ± 4.17	6.67 ± 6.01	2.02 ± 2.31	4.30 ± 4.92	ND	5.14 ± 5.89	8.12 ± 1.86	7.86 ± 3.89	ND
22	Aldehyde	5-Methyl-2-thiophenecarboxaldehyde	ND	ND	ND	ND	ND	ND	12.43 ± 3.37	18.11 ± 2.47	16.57 ± 3.29	8.65 ± 1.08	8.16 ± 1.48	10.14 ± 2.31	9.13 ± 1.85	ND	ND	ND	ND	ND	ND	ND
23	Aldehyde	4-Ethylbenzaldehyde	ND	ND	ND	ND	ND	ND	ND	ND	ND	ND	ND	ND	ND	1.25 ± 1.43	5.77 ± 5.00	9.84 ± 0.30	3.83 ± 3.32	0.64 ± 1.12	5.81 ± 5.50	13.05 ± 1.59
24	Alcohol	1-Penten-3-ol	ND	ND	ND	ND	536.85 ± 267.07	710.47 ± 245.37	2.77 ± 2.41	1.43 ± 2.48	2.71 ± 4.70	2.05 ± 1.85	2.16 ± 1.87	7.61 ± 0.82	10.42 ± 1.58	4.53 ± 0.84	2.43 ± 2.15	8.66 ± 0.71	5.73 ± 0.28	ND	9.89 ± 1.07	16.30 ± 1.14
25	Alcohol	2-Penten-1-ol (total isomers)	ND	ND	ND	ND	145.52 ± 131.37	140.28 ± 151.14	10.38 ± 1.67	8.71 ± 7.55	13.60 ± 1.59	8.30 ± 7.19	44.09 ± 10.81	3.30 ± 5.72	11.12 ± 0.96	3.75 ± 0.32	4.01 ± 1.09	5.58 ± 0.77	4.88 ± 0.50	6.60 ± 0.52	7.00 ± 0.39	12.16 ± 0.99
26	Alcohol	(Z)-1,5-Octadien-3-ol	ND	ND	ND	ND	52.68 ± 16.53	146.89 ± 76.28	18.79 ± 5.30	14.12 ± 2.26	24.51 ± 4.49	11.77 ± 0.68	13.41 ± 1.94	26.68 ± 4.89	41.92 ± 0.59	46.60 ± 0.82	33.07 ± 4.79	53.36 ± 0.83	38.22 ± 0.97	48.92 ± 2.73	54.30 ± 2.82	78.90 ± 4.91
27	Alcohol	2,7-Octadien-1-ol	ND	ND	ND	ND	53.07 ± 27.45	152.35 ± 17.41	24.23 ± 3.74	25.42 ± 5.19	35.98 ± 2.01	17.56 ± 2.08	16.53 ± 1.42	31.83 ± 6.81	60.90 ± 11.07	24.69 ± 2.66	22.91 ± 2.44	32.25 ± 1.01	27.28 ± 0.84	32.85 ± 2.51	36.56 ± 0.62	79.67 ± 6.14
28	Alcohol	1-Octen-3-ol	ND	ND	1.38 ± 2.39	0.48 ± 0.84	ND	ND	8.06 ± 2.13	7.75 ± 1.28	10.83 ± 4.45	4.87 ± 1.93	6.50 ± 0.70	6.02 ± 1.64	4.30 ± 3.77	5.21 ± 0.58	4.43 ± 0.63	9.82 ± 0.53	7.91 ± 0.34	6.96 ± 0.70	6.10 ± 0.74	ND
29	Alcohol	1-Octanol	ND	0.08 ± 0.01	2.10 ± 0.11	88.77 ± 2.75	ND	ND	ND	ND	ND	1.50 ± 2.60	28.61 ± 2.72	ND	ND	ND	0.89 ± 1.54	6.08 ± 1.32	8.37 ± 0.46	37.75 ± 2.15	2.73 ± 3.13	5.12 ± 5.87
30	Alcohol	1-Heptanol	ND	0.04 ± 0.07	4.38 ± 3.80	65.25 ± 2.90	ND	ND	1.87 ± 1.71	1.29 ± 2.24	3.87 ± 1.14	2.08 ± 1.80	28.61 ± 2.72	3.81 ± 1.73	4.84 ± 1.22	ND	1.40 ± 1.60	ND	6.46 ± 0.70	26.34 ± 2.68	ND	ND
31	Alcohol	1-Hexanol	ND	ND	50.87 ± 4.56	2.35 ± 0.40	ND	ND	ND	ND	ND	1.60 ± 1.39	28.61 ± 2.72	ND	ND	0.06 ± 0.07	ND	0.62 ± 0.23	11.88 ± 0.75	ND	0.04 ± 0.08	ND

32	Alcohol	Linalool	ND	ND	ND	ND	ND	ND	10.56 ± 1.26	10.70 ± 1.59	16.19 ± 1.11	10.20 ± 1.48	9.75 ± 1.65	9.40 ± 1.82	9.84 ± 0.80	ND	ND	ND	ND	ND	ND	ND
33	Alcohol	2-Octyn-1-ol	ND	ND	ND	ND	ND	ND	ND	ND	ND	ND	ND	ND	ND	3.52 ± 3.14	ND	14.52 ± 0.38	9.15 ± 2.40	5.89 ± 6.75	17.79 ± 0.82	34.01 ± 1.05
34	Ketone	1-Penten-3-one	ND	ND	ND	ND	186.43 ± 107.19	198.33 ± 104.59	ND	ND	ND	ND	ND	ND	ND	ND	ND	ND	ND	ND	ND	ND
35	Ketone	2-Octanone	ND	ND	19.80 ± 3.04	ND	ND	ND	ND	ND	ND	36.94 ± 34.83	ND	ND	ND	ND	ND	ND	62.38 ± 41.42	ND	ND	ND
36	Ketone	2-Nonanone	ND	0.05 ± 0.04	ND	2.30 ± 1.15	ND	ND	1.98 ± 3.42	ND	ND	0.56 ± 0.97	47.92 ± 2.50	1.47 ± 1.28	0.87 ± 1.50	ND	ND	ND	1.40 ± 1.21	40.68 ± 2.51	ND	ND
37	Ketone	3-Nonanone	ND	ND	ND	ND	ND	ND	ND	ND	ND	ND	ND	1.67 ± 1.45	1.32 ± 2.28	ND	1.05 ± 1.21	4.05 ± 0.29	0.36 ± 0.63	1.43 ± 1.63	2.41 ± 1.19	5.44 ± 0.62
38	Ketone	2-Decanone	ND	ND	1.22 ± 1.06	3.58 ± 0.48	ND	ND	ND	ND	ND	ND	ND	ND	ND	ND	ND	ND	ND	40.17 ± 3.73	ND	ND
39	Ketone	2-Tridecanone	1.91 ± 0.20	0.12 ± 0.11	ND	ND	ND	ND	ND	ND	ND	ND	ND	ND	ND	ND	53.12 ± 10.46	ND	ND	ND	ND	ND
40	Ketone	2-Pentadecanone	ND	ND	ND	ND	34.90 ± 10.17	ND	ND	ND	ND	ND	ND	ND	ND	ND	ND	ND	ND	ND	ND	ND
	Ketone	6-Octen-2-one (total isomers)		ND	ND	ND	74.48 ± 31.49	120.43 ± 26.87	10.12 ± 2.10	8.52 ± 1.70	12.22 ± 0.99	41	7.64 ± 0.63	14.38 ± 2.62	20.25 ± 0.65	4.68 ± 0.20	3.58 ± 3.17	6.89 ± 1.49	0.71 ± 1.23	6.46 ± 0.81	13.45 ± 0.34	21.71 ± 3.56
42	Ketone	3,5-Octadien-2-one (total isomers)	ND	ND	ND	0.27 ± 0.47	555.34 ± 281.47	830.28 ± 234.42	2.54 ± 4.40	13.56 ± 11.74	21.13 ± 3.50	12.12 ± 2.73	7.27 ± 6.37	14.09 ± 2.50	15.10 ± 13.76	4.28 ± 0.19	8.77 ± 1.00	10.03 ± 0.53	6.76 ± 1.19	7.54 ± 0.97	13.03 ± 5.83	11.61 ± 10.87
43	Ketone	6-Methyl-5-hepten-2-one	ND	ND	ND	ND	ND	ND	6.22 ± 10.7	10.45 ± 9.21	19.35 ± 1.86	13.79 ± 0.81	16.77 ± 1.40	6.30 ± 10.9	15.45 ± 0.30	0.79 ± 0.91	1.25 ± 2.17	ND	0.48 ± 0.42	0.61 ± 0.70	1.73 ± 0.86	ND

									8					2								
44	Ketone	Acetophenone	ND	ND	ND	ND	ND	ND	1.59 ± 1.40	3.61 ± 1.50	3.96 ± 0.44	3.65 ± 3.17	1.88 ± 1.63	1.22 ± 2.12	3.27 ± 2.87	ND	ND	ND	ND	ND	ND	ND
45	Ketone	4H-Pyran-4-one, 2-ethyl-3-hydroxy-	ND	ND	ND	ND	ND	ND	12.81 ± 1.95	ND	16.78 ± 14.53	9.20 ± 8.06	12.18 ± 0.99	13.36 ± 2.97	16.62 ± 1.32	ND	ND	ND	ND	ND	ND	ND
46	Furan/heterocycle	2-(2-Pentenyl)furan (total isomers)	ND	ND	ND	2.27 ± 3.93	295.57 ± 146.21	564.95 ± 139.48	17.77 ± 1.55	27.45 ± 3.85	23.60 ± 0.65	13.96 ± 2.99	12.55 ± 4.03	17.71 ± 3.70	31.18 ± 2.64	8.50 ± 1.38	13.42 ± 1.93	15.96 ± 0.81	7.70 ± 2.65	9.30 ± 1.12	15.84 ± 1.02	34.36 ± 1.31
47	Furan/heterocycle	2-Ethylfuran	ND	ND	ND	ND	326.49 ± 179.21	598.03 ± 376.45	ND	ND	ND	ND	ND	ND	ND	ND	ND	ND	ND	ND	ND	ND
48	Furan/heterocycle	2-Pentylfuran	ND	ND	1.09 ± 1.88	1.30 ± 0.25	ND	ND	ND	ND	ND	1.45 ± 1.41	ND	ND	ND	ND	ND	ND	0.56 ± 0.64	5.79 ± 2.49	ND	ND
49	Furan/heterocycle	2-Hexylfuran	ND	ND	6.58 ± 0.81	0.50 ± 0.86	ND	ND	ND	ND	ND	ND	ND	ND	ND	ND	ND	ND	ND	ND	ND	ND
50	Furan/heterocycle	2-Heptylfuran	ND	ND	1.54 ± 1.34	3.46 ± 0.41	ND	ND	ND	ND	ND	ND	ND	ND	ND	ND	ND	ND	ND	ND	ND	ND
51	Furan/heterocycle	2-Furanmethanol	ND	ND	ND	ND	ND	ND	ND	4.08 ± 1.37	2.64 ± 2.30	4.40 ± 0.33	ND	3.42 ± 0.37	3.39 ± 1.13	1.98 ± 0.02	4.72 ± 0.67	3.45 ± 0.30	4.78 ± 0.40	6.64 ± 0.96	3.63 ± 0.33	3.38 ± 0.64
52	Acid	Hexanoic acid	0.11 ± 0.18	0.18 ± 0.03	43.81 ± 1.93	7.58 ± 1.05	ND	ND	ND	ND	ND	6.07 ± 1.16	1.84 ± 1.61	ND	ND	ND	ND	ND	ND	ND	ND	ND
53	Acid	Heptanoic acid	0.26 ± 0.02	0.16 ± 0.03	222.19 ± 14.20	48.64 ± 8.44	14.16 ± 7.25	34.28 ± 8.99	4.31 ± 1.31	8.23 ± 0.26	8.56 ± 0.48	36.31 ± 2.91	7.70 ± 2.15	3.33 ± 1.04	6.70 ± 0.49	ND	0.89 ± 1.54	ND	24.36 ± 0.36	2.82 ± 1.35	ND	ND
54	Acid	Octanoic acid	1.58 ± 0.06	0.25 ± 0.06	216.40 ± 15.72	106.01 ± 24.20	8.53 ± 14.78	24.86 ± 35.70	4.37 ± 0.93	9.11 ± 2.52	10.00 ± 0.36	58.70 ± 4.84	15.77 ± 1.36	4.29 ± 0.71	4.94 ± 0.99	ND	2.81 ± 2.52	3.15 ± 0.35	44.59 ± 0.57	12.41 ± 1.12	1.83 ± 0.64	ND

55	Acid	Nonanoic acid	0.87 ± 0.15	0.31 ± 0.06	13.22 ± 4.18	56.10 ± 16.75	15.75 ± 7.52	12.11 ± 10.72	9.67 ± 0.40	15.97 ± 0.75	15.07 ± 0.57	14.04 ± 1.44	14.16 ± 1.12	7.89 ± 2.10	10.20 ± 1.31	1.35 ± 0.37	4.62 ± 2.90	2.24 ± 0.16	4.80 ± 0.27	7.85 ± 0.96	3.12 ± 0.48	3.30 ± 0.16
56	Acid	Decanoic acid	2.07 ± 0.54	0.49 ± 0.16	4.33 ± 0.57	3.33 ± 1.28	ND	8.87 ± 15.36	2.69 ± 0.36	7.49 ± 1.13	3.52 ± 3.05	5.98 ± 0.41	1.54 ± 2.67	2.98 ± 0.28	2.55 ± 0.58	ND	4.72 ± 1.69	0.40 ± 0.46	2.55 ± 0.02	ND	0.46 ± 0.53	ND
57	Amine	Trimethylamine	ND	ND	ND	ND	ND	ND	49.60 ± 10.59	61.87 ± 14.57	74.72 ± 9.71	75.43 ± 16.40	77.51 ± 36.18	86.90 ± 8.40	106.80 ± 19.64	33.91 ± 3.92	63.20 ± 12.30	60.65 ± 12.87	63.15 ± 4.68	53.83 ± 10.30	92.39 ± 13.79	73.63 ± 31.34

Notes:

1. Values are reported as mean ± SD from three independent preparation/heating replicates.

2. A compound was considered reproducibly detected when it was detected in at least two of three independent replicates. ND indicates detection in fewer than two of three replicates.

3. FA-only concentrations are expressed as μg/kg fatty acid based on the actual fatty acid mass used for HS-SPME-GC–MS analysis (0.025 g). LFK and KM concentrations are expressed as μg/kg sample based on a 5.00 g sample mass. Because the FA-only and matrix-containing systems use different concentration-normalization bases, absolute concentration magnitudes should not be compared directly across model tiers.

4. FA-only, fatty-acid-only model; LFK, sample code for the lipid-depleted krill matrix; KM, krill meat matrix.

Table S4. Odor thresholds and odor activity values of volatile compounds across the 20 thermal oxidation model groups.

No.	Chemical class	Compound	Odor threshold	FA-only model						LFK + FA model							KM + FA model						
			(µg/kg)	C14:0	C16:0	C16:1	C18:1	EPA	DHA	LFK	LFK+C14:0	LFK+C16:0	LFK+C16:1	LFK+C18:1	LFK+EPA	LFK+DHA	KM	KM+C14:0	KM+C16:0	KM+C16:1	KM+C18:1	KM+EPA	KM+DHA
1	Aldehyde	(E)-2-Decenal	0.3[1]	–	–	37.07	2941.14	91.84	–	–	15.56	9.48	4.36	22.41	–	8.52	0.6	4.15	6.85	12.9	21.12	–	0.97
2	Alcohol	1-Penten-3-ol	0.4[2]	–	–	–	–	1342.13	1776.17	6.93	3.57	6.79	5.12	5.41	19.02	26.05	11.33	6.06	21.64	14.32	–	24.74	40.74
3	Aldehyde	(6Z)-6-Nonenal	1[3]	–	–	907.07	43.34	–	120.09	–	–	–	–	–	–	–	–	–	–	9.41	2.17	–	–
4	Aldehyde	Nonanal	1[1]	0.39	1.86	4.55	298.85	18.22	–	52.97	47.44	65.28	51.92	128.03	47.22	37.07	21.04	28.31	37.12	36.98	101.81	35.76	36.23
5	Furan/heterocycle	2-Ethylfuran	2.3[1]	–	–	–	–	141.95	260.02	–	–	–	–	–	–	–	–	–	–	–	–	–	–
6	Aldehyde	Octanal	0.7[1]	9.02	0.21	24.31	217.38	–	–	17.36	–	21.29	7.84	104.39	–	–	3.09	8.12	13.88	5.26	33.31	7.01	0.27
7	Ketone	1-Penten-3-one	0.94[4]	–	–	–	–	198.33	210.99	–	–	–	–	–	–	–	–	–	–	–	–	–	–
8	Aldehyde	2-Octenal	3[1]	–	–	206.74	2.4	0.67	–	–	–	–	–	–	–	–	–	–	–	–	–	–	–
9	Aldehyde	(E,E)-2,4-Heptadienal	15.4[1]	–	–	–	–	167.25	196.73	–	–	0.26	–	–	0.24	0.32	0.41	0.61	1.01	0.83	–	1.11	0.36
10	Aldehyde	(2E,6Z)-2,6-Nonadienal	0.8[1]	–	–	–	0.27	171.04	9.69	4.2	6.73	13.58	3.21	3.29	7.66	15.37	3.56	5.6	10.37	6.26	7.39	9.93	19.19
11	Aldehyde	2-Undecenal	5[5]	–	–	1.64	157.4	4.42	0.78	–	–	–	–	–	–	–	–	–	–	–	–	–	–

12	Aldehyde	Heptanal	2.8[1]	–	–	31.42	19.18	–	–	1.36	5.73	–	–	8.19	6.32	–	0.82	0.54	0.95	–	–	3.11	–
13	Aldehyde	Decanal	2[1]	0.09	0.17	0.68	20.92	–	–	4.03	–	–	–	7.07	–	–	–	1.87	–	–	2.34	–	–
14	Aldehyde	Hexanal	5[3]	–	–	15.91	2.49	–	–	1.21	–	0.95	5.58	3.63	1.07	0.27	0.42	0.37	0.74	6.41	1.96	–	0.31
15	Alcohol	(Z)-1,5-Octadien-3-ol	10[3]	–	–	–	–	5.27	14.69	1.88	1.41	2.45	1.18	1.34	2.67	4.19	4.66	3.31	5.34	3.82	4.89	5.43	7.89
16	Alcohol	1-Heptanol	5.4[1]	–	<0.01	0.81	12.08	–	–	–	–	–	0.28	5.3	–	–	–	0.26	–	1.2	4.88	–	–
17	Amine	Trimethylamine	9[3]	–	–	–	–	–	–	5.51	6.87	8.3	8.38	8.61	9.66	11.87	3.77	7.02	6.74	7.02	5.98	10.27	8.18
18	Aldehyde	(Z)-4-Heptenal	4.2[1]	–	–	–	–	–	–	3.66	4.12	2.86	2.52	2.63	6.12	9.77	3.84	3.12	5.07	3.48	4.03	6.09	8.61
19	Alcohol	1-Octen-3-ol	1.5[1]	–	–	0.92	0.32	–	–	5.37	5.17	7.22	3.25	4.33	4.01	2.87	3.47	2.96	6.55	5.28	4.64	4.07	–
20	Ketone	3,5-Octadien-2-one	150[6]	–	–	–	<0.01	3.7	5.54	0.02	0.09	0.14	0.08	0.05	0.09	0.1	0.03	0.06	0.07	0.05	0.05	0.09	0.08
21	Ketone	2-Octanone	50[3]	–	–	0.4	–	–	–	–	–	–	0.74	–	–	–	–	–	–	1.25	–	–	–

Notes:

1. OAV was calculated as C_i/T_i, where C_i is the semi-quantitative concentration of a volatile compound and T_i is its odor threshold.

2. Published thresholds determined in water or food matrices were preferentially used. When only aqueous thresholds were available, values reported as ppb or µg/L in water were treated as approximately equivalent to µg/kg for screening-level OAV calculations.

3. OAVs below 0.01 are shown as <0.01. An en dash (–) indicates that the compound was not quantified in the corresponding group.

4. Because volatile concentrations were obtained by single-internal-standard semi-quantification, OAVs indicate potential odor relevance rather than absolute sensory contribution. Absolute OAV magnitudes should not be directly compared among the FA-only, LFK + FA, and KM + FA model tiers because these systems differ in composition, headspace partitioning, and concentration normalization. LFK denotes the sample code for the lipid-depleted krill matrix.

5. Superscript numbers refer to the threshold references listed below.

Threshold References

1. Ma, R.; Liu, X.; Tian, H.; Han, B.; Li, Y.; Tang, C.; Zhu, K.; Li, C.; Meng, Y. Odor-active volatile compounds profile of triploid rainbow trout with different marketable sizes. *Aquac. Rep.* **2020**, *17*, 100312. https://doi.org/10.1016/j.aqrep.2020.100312
2. Yimdee, T.; Wang, X.-C. Comparison of odor and taste of commercial brand fish sauces from East and South East Asian countries. *Int. J. Food Prop.* **2016**, *19*, 873–896. https://doi.org/10.1080/10942912.2015.1045517
3. van Gemert, L.J. *Odour Thresholds: Compilations of Odour Threshold Values in Air, Water and Other Media*, 2nd ed.; Oliemans Punter & Partners BV: Utrecht, The Netherlands, 2011.
4. Mall, V.; Sellami, I.; Schieberle, P. New degradation pathways of the key aroma compound 1-penten-3-one during storage of not-from-concentrate orange juice. *J. Agric. Food Chem.* **2018**, *66*, 11083–11091. https://doi.org/10.1021/acs.jafc.8b04334
5. Geng, H.; Chen, L.; Wang, S.; Wu, Y.; Gao, P.; Jiang, Q.; Yang, F.; Xia, W. Analysis of volatile compounds contributing to distinctive odour of silver carp (Hypophthalmichthys molitrix) surimi. *Int. J. Food Sci. Technol.* **2022**, *57*, 7774–7786. https://doi.org/10.1111/ijfs.16141
6. Sung, W.-C.; Lin, H.-T.; Liao, W.-C.; Fang, M. Effects of halogen lamp and traditional sun drying on the volatile compounds, color parameters, and gel texture of Gongliao Gelidium seaweed. *Foods* **2023**, *12*, 4508. https://doi.org/10.3390/foods12244508
